# The impact of the atmospheric turbulence-development tendency on new particle formation: a common finding on three continents

**DOI:** 10.1093/nsr/nwaa157

**Published:** 2020-07-06

**Authors:** Hao Wu, Zhanqing Li, Hanqing Li, Kun Luo, Yuying Wang, Peng Yan, Fei Hu, Fang Zhang, Yele Sun, Dongjie Shang, Chunsheng Liang, Dongmei Zhang, Jing Wei, Tong Wu, Xiaoai Jin, Xinxin Fan, Maureen Cribb, Marc L Fischer, Markku Kulmala, Tuukka Petäjä

**Affiliations:** State Key Laboratory of Remote Sensing Science, College of Global Change and Earth System Science, Beijing Normal University, Beijing 100875, China; ESSIC and Department of Atmospheric Science, University of Maryland, College Park, MD 21029, USA; State Key Laboratory of Clean Energy Utilization, Zhejiang University, Hangzhou 310027, China; State Key Laboratory of Clean Energy Utilization, Zhejiang University, Hangzhou 310027, China; School of Atmospheric Physics, Nanjing University of Information Science and Technology, Nanjing 210044, China; Meteorological Observation Center, China Meteorological Administration, Beijing 100081, China; State Key Laboratory of Atmospheric Boundary Layer Physics and Atmospheric Chemistry, Institute of Atmospheric Physics, Chinese Academy of Sciences, Beijing 100029, China; State Key Laboratory of Remote Sensing Science, College of Global Change and Earth System Science, Beijing Normal University, Beijing 100875, China; State Key Laboratory of Atmospheric Boundary Layer Physics and Atmospheric Chemistry, Institute of Atmospheric Physics, Chinese Academy of Sciences, Beijing 100029, China; State Key Joint Laboratory of Environmental Simulation and Pollution Control, College of Environmental Sciences and Engineering, Peking University, Beijing 100871, China; State Key Joint Laboratory of Environment Simulation and Pollution Control, School of Environment, Tsinghua University, Beijing 100084, China; State Key Laboratory of Remote Sensing Science, College of Global Change and Earth System Science, Beijing Normal University, Beijing 100875, China; State Key Laboratory of Remote Sensing Science, College of Global Change and Earth System Science, Beijing Normal University, Beijing 100875, China; State Key Laboratory of Remote Sensing Science, College of Global Change and Earth System Science, Beijing Normal University, Beijing 100875, China; State Key Laboratory of Remote Sensing Science, College of Global Change and Earth System Science, Beijing Normal University, Beijing 100875, China; State Key Laboratory of Remote Sensing Science, College of Global Change and Earth System Science, Beijing Normal University, Beijing 100875, China; ESSIC and Department of Atmospheric Science, University of Maryland, College Park, MD 21029, USA; Lawrence Berkeley National Laboratory, Berkeley, CA 94720, USA; Institute for Atmospheric and Earth System Research / Physics, Faculty of Science, University of Helsinki, Helsinki 00014, Finland; Aerosol and Haze Laboratory, Beijing Advanced Innovation Center for Soft Matter Science and Engineering, Beijing University of Chemical Technology, Beijing 100029, China; Joint International Research Laboratory of Atmospheric and Earth System Sciences, School of Atmospheric Sciences, Nanjing University, Nanjing 210023, China; Institute for Atmospheric and Earth System Research / Physics, Faculty of Science, University of Helsinki, Helsinki 00014, Finland; Joint International Research Laboratory of Atmospheric and Earth System Sciences, School of Atmospheric Sciences, Nanjing University, Nanjing 210023, China

**Keywords:** new particle formation, turbulence development, molecular dynamic

## Abstract

A new mechanism of new particle formation (NPF) is investigated using comprehensive measurements of aerosol physicochemical quantities and meteorological variables made in three continents, including Beijing, China; the Southern Great Plains site in the USA; and SMEAR II Station in Hyytiälä, Finland. Despite the considerably different emissions of chemical species among the sites, a common relationship was found between the characteristics of NPF and the stability intensity. The stability parameter (ζ = *Z*/*L*, where *Z* is the height above ground and *L* is the Monin–Obukhov length) is found to play an important role; it drops significantly before NPF as the atmosphere becomes more unstable, which may serve as an indicator of nucleation bursts. As the atmosphere becomes unstable, the NPF duration is closely related to the tendency for turbulence development, which influences the evolution of the condensation sink. Presumably, the unstable atmosphere may dilute pre-existing particles, effectively reducing the condensation sink, especially at coarse mode to foster nucleation. This new mechanism is confirmed by model simulations using a molecular dynamic model that mimics the impact of turbulence development on nucleation by inducing and intensifying homogeneous nucleation events.

## INTRODUCTION

New particle formation (NPF) has been observed worldwide, chiefly occurring in the planetary boundary layer (PBL) [1–4]. Particle nucleation begins with the clustering of precursor gas molecules to form the embryos of particles, leading to the formation of secondary aerosols in the atmosphere [5,6]. These aerosols may further grow through condensation and coagulation to become cloud condensation nuclei [7–9] to affect weather and climate, besides causing severe air-pollution episodes, especially in densely populated regions like China [10–13]. The chemical processes involved in NPF have been the focus of many previous studies. The key chemical species that form stable clusters remain uncertain, differing from region to region and from time to time [14–16], including H_2_SO_4_, amines and organic acids. While the photochemical processes have been regarded as the underlying mechanisms leading to the onset and growth of the NPF [17–20], some physical processes may also play important roles in the NPF, such as radiation, cloudiness, aerosol surface area and aerosol dynamics in the boundary layer [21–23]. Most recently, it has been shown that automobile emissions constitute an important source for NPF under urban conditions, mainly from photochemical oxidation of aromatic organic compounds [24], but it remains an open question as to their relative importance.

Atmospheric stability influences the mixing of gases and particles. As a result, turbulent flows can impact the evolution and spatial distribution of aerosol precursor gases and pre-existing particles and thus the NPF. However, no solid physical mechanism has been established to explain the relationship between NPF and turbulence development. Laboratory experiments have been conducted using different chambers to investigate NPF processes [25–27]. Recent aircraft measurements showed that PBL development and vertical mixing can promote ultrafine particle bursts in the residual layer [28] and are highly associated with NPF [29,30]. Analyses of the development of the PBL and the NPF have shown that random and sudden mixing processes in a thermally unstable atmosphere are favorable for particle nucleation [31,32]. Turbulence in a well-mixed boundary layer may cause temperature fluctuations and increase the upward motion of atmospheric components, which may favor NPF [33,34]. Its impact is, however, tangled with many other influential factors, such as the condensation sink (CS) provided by pre-existing particles. In polluted environments, there is a positive feedback between the aerosol concentration and the boundary-layer mixing [35]. However, the connections and feedback between the mixing process in connection with the NPF have not been fully understood [36–40].

Using the Lennard-Jones model, Yasuoka and Zeng [41] investigated the effect of turbulence on nucleation events and found a positive interaction between particles and the carrier-gas pressure at the cluster surface. A vapor–liquid–solid model was built to simulate the growth of single-walled carbon nanotubes at different temperatures and varying supersaturation levels [42]. Dzwinel *et al.* [43] found that Rayleigh–Taylor instability connects turbulent motion from the macroscopic world to the molecular scale. The bubble-and-spikes stage of the mixing process is similar. Supersaturation renders ultra-high pressure to allow gaseous molecules to either cluster together or break into a liquid/solid barrier to form new particles [44–46]. Other molecular approaches have been taken to understand the mechanism [47,48], some of which have been applied to particle nucleation [49–51]. Olenius *et al.* [52] proposed that a stochastic effect exists in nanoparticle growth and found that sink scavenging may increase the particle diameter. They attributed it to the decreases in the evaporation of existing particles by considering the interaction between the particle-phase volume fraction and the fluid-phase velocity fluctuations. This helps to explain why NPF occurs in an unstable layer, where new mixing production causes a granular temperature gradient when the upstream boundary of the cluster velocity field is highly compressed [53]. Under such a circumstance, fluctuation appears and facilitates more collisions, facilitating nucleation to occur more easily, as was shown by Monte-Carlo simulations [54,55].

This study first investigates the impact of the turbulence-development tendency on the NPF process based on ample observations from stations on three continents (China, the USA and Finland; cf. SI 1.1 and Supplementary Fig. 1) with distinct environments. Due to the heterogeneity of the environments, it is plausible to assume that precursors such as sulfuric acid or their combinations would explain the NPF events. The stability parameter (ζ = *Z*/*L*, where *Z* is the height above ground and *L* is the Monin–Obukhov length), however, is found to play a common role, fluctuating significantly before NPF. This feature may serve as an indicator of nucleation bursts. Differently from the above simulation studies that used large-scale models or kinetic equations, we then applied a microscale molecular-dynamics simulation model to investigate how turbulence influences the temperature distribution that finally affects the physical process of NPF. Finally, a possible physical mechanism is proposed and quantified.

## RELATIONSHIP BETWEEN NPF EVENTS AND THE TURBULENCE TENDENCY

**Figure 1.**
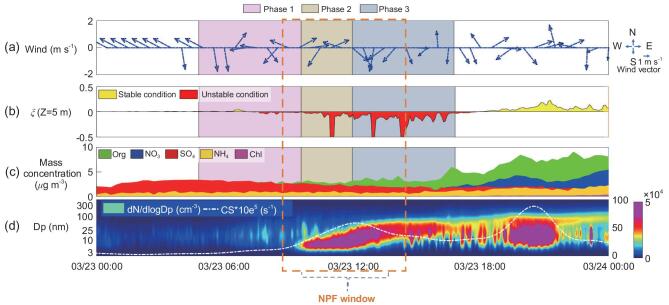
Evolutions of a typical regional spring NPF event (23 March 2019) and associated variables in Beijing. (a) 1-h average wind vector. Arrows represent the wind direction and their lengths show the wind speed. (b) The stability parameter (ζ). (c) The aerosol mass concentrations of chemical species. (d) The particle-number size distribution. The white dashed curve shows CS × 10e^5^. The shaded areas mark different periods during the daytime: Phase 1, prior to the NPF; Phase 2, the initiation of NPF; Phase 3, the growth of the NPF event. The duration of an NPF event is the total period of Phases 2 and 3, and the dash box is the NPF window (12:00 ± 3:00).

During a series of field experiments aimed at monitoring and understanding aerosols and their impact on clouds, precipitation and the climate, a large number of instruments were deployed in Beijing (Supplementary Fig. 2), northern China from 2013 [11]. Measurements show that the NPF events in spring and autumn occur more frequently than in summer, which is consistent with the previous finding [56]. The instruments include the scanning mobility particle sizer model 3938 (TSI Inc., USA) that acquires the particle-number size distribution (PNSD) from 11.3 to 552.3 nm in 5 min in 64 channels; the eddy covariance 7500A system (LI-COR Biosciences, Inc., USA); the WindMaster 3D anemometer (R3–50, Gill Instruments Limited, UK) that measures the wind in 3D and characterizes turbulent features [57–59]; and the quadrupole aerosol chemical speciation monitor (Aerodyne Research, Inc., USA) that measures aerosol chemical species, among many others (cf. SI 1.2). The duration of an NPF event in this study was measured from the time at which the concentration of the nucleation mode (<25 nm) suddenly increased until no obvious growth at the end of that day or when the concentration dropped sharply [60]. The NPF window is defined as the period of 3 hours before and after midday (12:00 ± 3:00). Figure 1 shows the time series of a regional NPF episode and related variables observed at the Beijing site.

NPFs are identified when the maximum concentration of particles <25 nm in diameter is >3 × 10^4^ cm^−3^ (excluding the background-noise concentration) and particle growth was sustained for several hours [61], after excluding the noise induced by changes in wind direction linked with emissions, especially those from the nearby major road, the 5^th^ Ring Road in Beijing (cf. SI 1.3). A typical NPF event occurred at around 10:30 local time (LT) on 23 March 2019 (Phase 2 in Fig. 1) and then grew into the accumulation mode (Phase 3), ending at around 16:00 LT. The stronger wind from the north likely reduced the pre-existing particles to create a clean regime during Phase 1 (Fig. 1a). Stability fluctuations are also shown in Phase 2 when the atmosphere became unstable (Fig. 1b). The large negative values of ζ mean strong turbulence. The mass concentration of organics (green area in Fig. 1c) grew sharply after the NPF bursts, indicating that the organics were transformed by reactions between volatile organic compounds, and then condensed into the particle phase (Fig. 1c; more information in SI 1.4).

Based on all NPF events that occurred in Beijing during the study period (Supplementary Table 2), the influences of the atmospheric stability on the process of NPF are divided into three phases:

- Phase 1. High-speed wind from the north diluted the pre-existing particles (Fig. 1a) and reduced the CS to its lowest level, creating a favorable environment for NPF when the atmosphere became more unstable (Fig. 1b).
- Phase 2. Once the wind speed diminished (Fig. 1a) and the atmospheric instability was further strengthened (Fig. 1b), the NPF started showing in the PNSD (Fig. 1d).
- Phase 3. The wind speed showed no significant changes and the atmosphere stabilized (Fig. 1b). NPF terminated at night, particles grew to >100 nm and the mass concentration increased, especially that of organic compounds (Fig. 1c). At night, traffic emissions occupied a greater portion of the PNSD (Supplementary Fig. 3).

Once the surface layer had transitioned to an unstable regime, the CS (white dashed line in Fig. 1d) showed a synchronal decreasing trend, accompanied by a burst in the nucleation-mode concentration. The nucleated particles continued to grow, causing the ‘banana shape’ of an NPF to appear in the PNSD. This implies that enhancing the tendency toward turbulence development can impact the gas–solid phase transformation process. Based on the NPF events and the variation in ζ, two general cases are considered: (i) strengthening turbulence—the stability parameter dropped below the threshold of –0.5 in Phase 2 (ζ < –0.5, increasing in its magnitude $|$ζ$|$) and (ii) weakening turbulence—the stability parameter was within the –0.5 threshold in Phase 2 (–0.5 < ζ < 0). We find that it is the stability parameter, rather than the turbulence intensity itself (turbulent kinetic energy, Richardson number or others), that impacts the NPF the most (Supplementary Fig. 4). Therefore, we chose to use ζ to scale the turbulence tendency in the surface layer and considered both stable and unstable conditions. The ζ showed a significant downward trend before the NPF event compared to that on a non-event day (other in Supplementary Fig. 5), demonstrating the potential impact of the turbulence tendency on the occurrence of NPF.

The relationship between **ζ** and NPF key factor CS was calculated (for other modes, cf. SI 1.4) for all NPF events that occurred under strengthening-turbulence conditions and it is found that the correlation between **ζ** and CS reached 0.73 at Beijing (duration in Supplementary Fig. 6). For weakening turbulence, however, the correlation was not obvious. Under these conditions, particle growth could not be sustained any longer and ended earlier compared to that during strong turbulence. This implies that stronger solar radiation on an event day controlled the turbulence development tendency (Supplementary Figs 7 and 8), influencing the evolution of the PNSD in the PBL [62]. The hypothesis behind this mechanism is that increased unstable stratification generates high local supersaturation levels, fostering NPF as in the case of the formation of cloud droplets [45]. Strengthening turbulence can create an environment that promotes gas-to-particle conversion during the nucleation process due to the preferential concentration effect [63,64]. On the other hand, decreases in the CS also facilitate the growth process. However, in some cases, NPF did not happen when the CS was at a low level or when the turbulence was strong, but no obvious decrease in the CS was seen (Supplementary Fig. 9). A possible explanation is that particles larger than the accumulation mode (>100 nm) may cause some feedback that results in intermittent turbulence [65]. We also analysed to gain further insight into the relationship between NPF occurrence and the stability intensity. Strengthening-turbulence events (i.e. NPF presented by stability parameter ζ < –0.5) were more common than weakening-turbulence events at these stations (Supplementary Fig. 10). Table 1 summarizes the statistics at each site.

**Table 1.** The total number of days with NPF events at each site and the percentage of events satisfying the criteria describing the cases.

Location	Beijing	SGP	Hyytiälä
Duration	July–August, 2017	April–May, 2013	March–April, 2013
Site description	Megacity	Grassy plain	Boreal forest
Days for observation	19	33	38
Days for NPF	8	11	20
Percentage of cases with strong fluctuations before NPF	62.5%	72.7%	80%
Percentage of cases with weak fluctuations before NPF	37.5%	27.3%	20%

**Figure 2.**
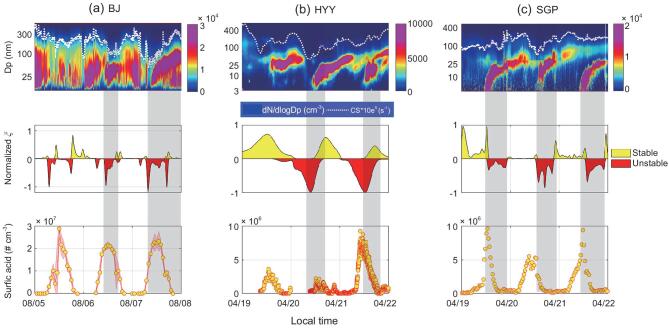
The evolutions of typical NPF events in Beijing (BJ) in China (a), Hyytiälä (HYY) in Finland (b) and the Southern Great Plain (SGP) in the USA (c). Upper panels: the PNSD with the white line showing CS*10e^5^. Mid panels: the associated changes in normalized stability parameter ζ. Lower panels: sulfuric acid.

Figure 2 presents the measurements of particle-number size distributions from which NPF events are identified as marked in shaded areas and the associated evolutions of CS, turbulence and sulfuric acid in the three continents: Beijing (BJ) (a), Hyytiälä (HYY) (b) [66,67] and the South Great Plains (SGP) (c). At all three sites, the evolutions are largely in concert with NPF, normalized ζ and CS. In comparison, coherences between the evolutions of NPF and sulfuric acid vary among the stations. In Beijing and SGP, there were strong diurnal variations, presumably because of photochemical processes and the NPFs that occurred shortly after the peaks, whereas this is not the case in HYY, where NPF happened when the sulfuric acid was both low and high but turbulence was persistently strong. This implies that chemical composition is not the sole cause of NPF and that turbulence played a key role.

**Figure 3.**
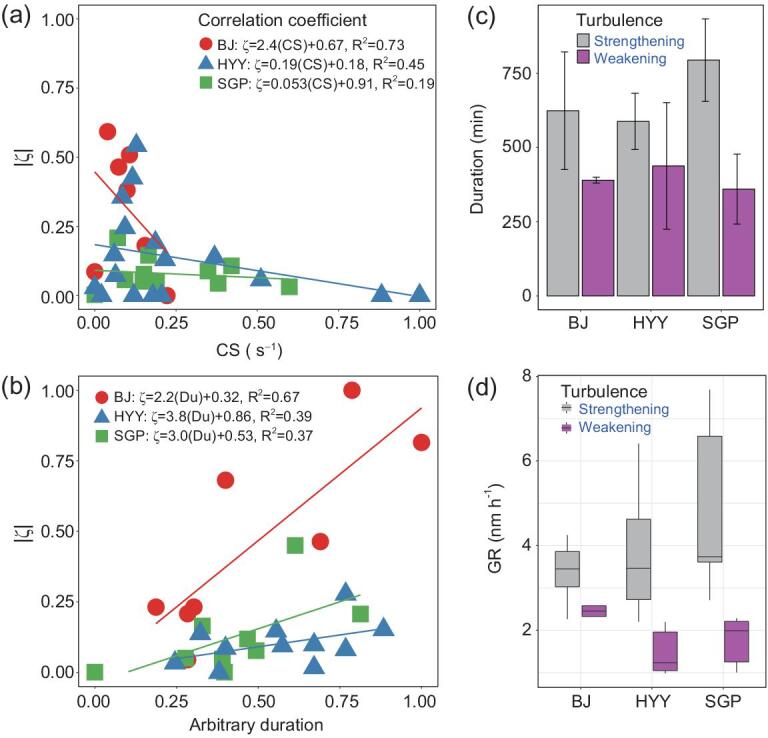
(a) Correlation between the normalized condensation sink in Phase 1 and the atmospheric stability intensity ($|$ζ$|$). (b) The $|$ζ$|$ as a function of the normalized NPF duration at the Beijing (BJ, red circles), Hyytiälä (HYY, blue triangles) and the US Southern Great Plain (SGP, green squares) sites (cf. SI 4.2). (c) NPF duration and (d) comparison of the growth rates (GR) of cases with strengthening turbulence (gray) and weakening turbulence (magenta) at the three sites.

To further understand how turbulence tendency influences the NPF growth process, we analysed the dependence of CS (Fig. 3a and Supplementary Figs 11–13) and the normalized (*x* = (*x* – *u*)/σ) NPF duration (Fig. 3b), as well as the mean durations (Fig. 3c) and growth rates (Fig. 3d) of NPF under strengthening and weakening turbulence. There is a good correlation between $|$ζ$|$ and the CS variation. The shape of the fitted line indicates that stronger turbulence enhances the dilution of pre-existing particles, lowering the sink effect. The CS changed rapidly when ζ decreased sharply, implying that an increasing ζ depresses the CS, facilitating NPF by preventing small particles from being scavenged on the surface of pre-existing particles. There is also a positive-correlation property especially at Beijing between the stability parameter and the duration of an NPF event (Fig. 3b). Turbulence-strengthening processes seem to prolong the duration of NPF, which is consistent with the evolution of the turbulence tendency and nucleation mode particle bursting. The NPF duration is systematically longer under strengthening-turbulence than under weakening-turbulence conditions (Fig. 3c). The growth rate is faster under strengthening-turbulence conditions (>3.2 nm h^−1^) than under weakening-turbulence conditions (<2.4 nm h^−1^) (Fig. 3d).

It appears that stability intensity is a prominent factor impacting particle growth (Supplementary Fig. 14). Once the vapors overcome the energy barrier promoted by turbulence evolution, a spontaneous particle formation occurred. Under certain circumstances, however, turbulence development may limit NPF because the coagulation of small particles onto larger ones could increase the surface areas of the larger particles, thus strengthening the CS for ultrafine particles.

## EXPLORING THE IMPACT OF THE TURBULENCE-DEVELOPMENT TENDENCY ON NPF USING A MOLECULAR-DYNAMICS MODEL

An NPF event begins with the clustering of thermodynamically stable molecules, creating nanoparticles consisting of embryos that grow quickly without being scavenged by the coagulation sink through collisions with larger particles [68]. To verify our hypotheses concerning the impact of turbulence development on both the initial nucleation and coagulation, we conducted numerical simulations using a microscale molecular-dynamics model instead of the general dynamic equation typically used in other studies [69]. In our simulations, the reactive force field [70] was used to demonstrate interatomic interactions. The simulation was conducted in a nanoscale periodic box with a domain of 32 nm × 32 nm × 120 nm. The canonical ensemble [71] was adopted, and the Berendsen thermostat [72] with a 0.1-ps damping constant, was used to correct the temperature. The simulation time step was chosen as 0.25 fs. Because of the limitation of space, turbulence structures are difficult to express in a microscale simulation and we assume that the kinetic energy of turbulence had been dissipated into internal energy and was manifested as a non-uniform regional temperature field. The macroscopic momentum of the gas field within the calculation space (length = 0.1 μm) was almost uniform. Table 2 summarizes the temperature fields in the simulation domains. The standard deviation (σ) is used to represent the temperature fluctuations.

**Table 2.** Simulation settings.

Turbulence level	Case	Average temperature	σ
No turbulence	No perturbation	290 K	0
Low turbulence	Low perturbation	290 ± 5 K	1.02%
High turbulence	High perturbation	290 ± 15 K	3.06%

**Figure 4.**
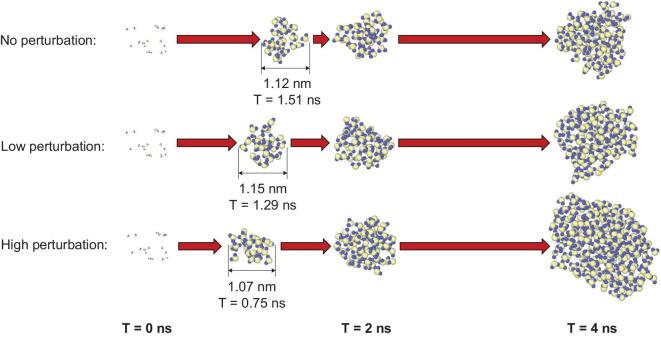
Evolution of the largest particles in simulations under different turbulence conditions.

In an experimental study, a particle size of 1.1–1.9 nm is usually considered a minimum size for the critical point in the NPF process. Particles under this critical size have direct connection to NPF [73]. A particle with a diameter of 1.1 nm consists of ∼24 H_2_O molecules. Here we use water molecules as virtual molecules to mimic atmospheric clustering, although, in reality, sulfuric acid and amines/ammonia are clustering. Figure 4 illustrates the evolution process of the largest particle under different turbulence conditions. The moment of the generation of the first particles containing 24 molecules and their sizes are given for each turbulence condition. It is evident that the higher the turbulence, the faster the particle grows.

**Figure 5.**
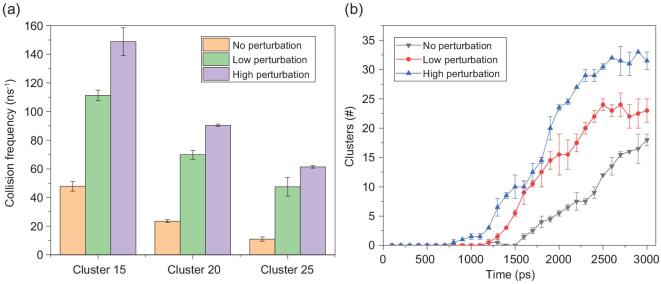
(a) The collision frequencies of three types of clusters and (b) the number of particles with diameters greater than the critical size.

The enhancement of collisions between three types of clusters (clusters containing more than 10, 15 and 25 H_2_O molecules) under different turbulence conditions is further shown in Fig. 5a. It is found that the collision frequency under high-turbulence conditions can be 3-fold higher than that without turbulence. Fluxes in the pre-nucleation cluster are expressed as the product of the turbulent diffusion coefficient and the gradient of a mean quantity because the Brownian diffusion caused by thermal motion controls all small molecules. The evolution of the 20 largest particles suggests that the influence of turbulence on the number of particles containing >24 H_2_O molecules is also visible, as shown in Fig. 5b. These results imply that, in a physical regime with no barriers, two molecules from different sources can meet to form a dimer. A two-molecule cluster may also evaporate and transform back into its monomers at a lower rate or collide with a third molecule to form a trimer. The presence of a cluster suggests that this physical process is influenced by turbulence.

Beyond the microscale, NPF can also be affected by turbulence in two ways. First, stronger turbulence generates supersaturation, which decreases the Gibbs free energy, allowing clusters to overcome the energy barrier and proceed to the particle phase faster. This is similar to Wehner's finding that turbulence likely leads to the supersaturation required for the nucleation of possible precursor gases [36]. Second, surface turbulence can bring clear air into the particle regime and dilute the regime between newly formed particles and pre-existing particles. During this process, CSs decrease significantly, indicating that, once unstable conditions are triggered, particles grow more effectively. Clusters will then be compressed and can overcome the kinetic-energy barrier to form a particle. Due to turbulent diffusion, strong coherent structures of dilution effectively segregate pre-existing particles, which also exerts an influence on the particle-size distribution, thus favoring the growth of nucleated particles.

## NEW MECHANISM OF THE IMPACT OF TURBULENCE ON NPF

**Figure 6.**
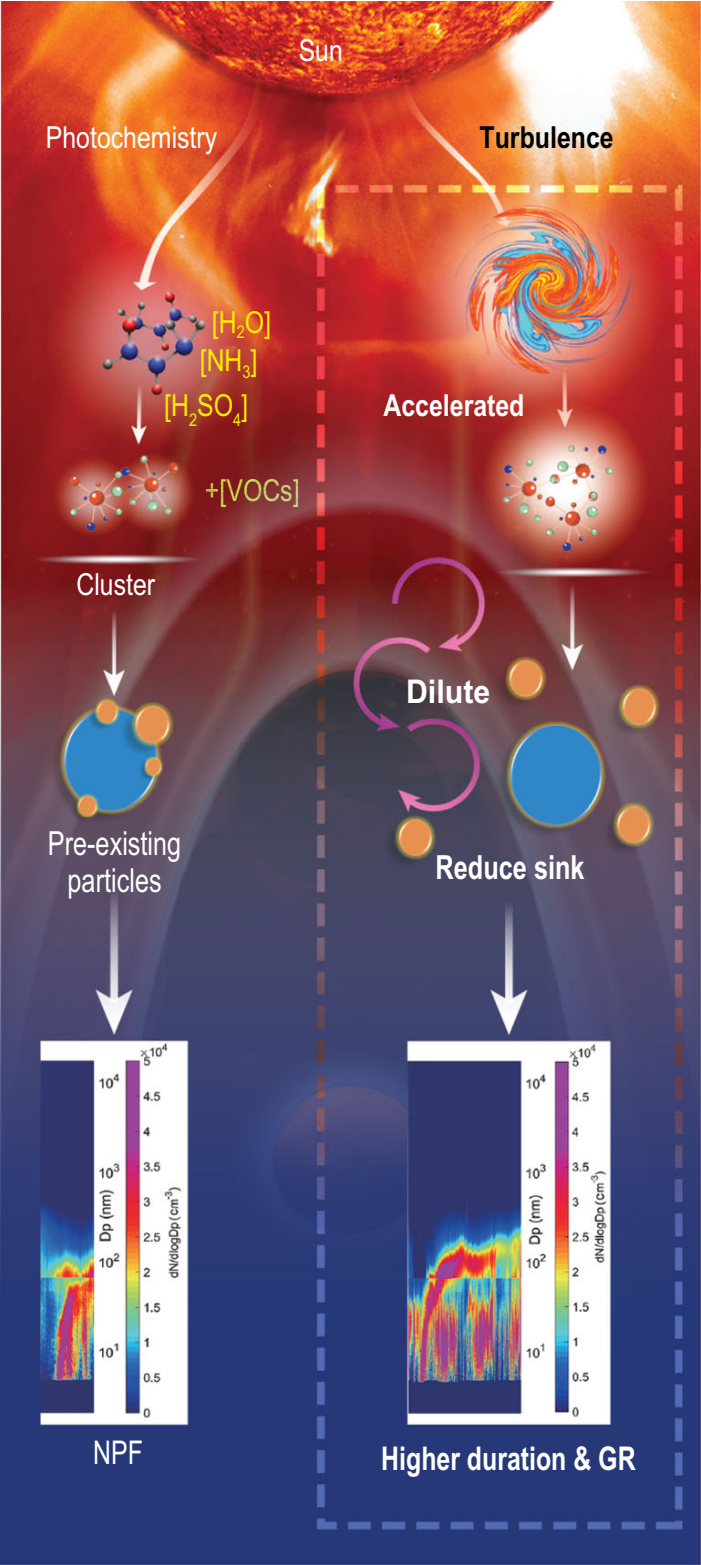
Schematic illustration of the impact of turbulence on the nucleation, duration and growth rate of a new particle formation process. The upper part illustrates the nucleation process at the nano scale, which describes the condensation of precursor gas molecules to form a cluster to the critical size (1.1–1.9 nm). Turbulence may enhance supersaturation to accelerate this process. When it grows to the atmospheric scale, the newly formed particles could be easily scavenged by pre-existing particles without turbulence, resulting in short and slow growth. With the turbulence-diluting effect, the pre-existing particles decrease, leading to lower coagulation sink, and prolonging the fast growth of NPF.

Based on the above observation analyses and model simulations, the new mechanism of the impact of turbulence on NPF is illustrated in Fig. 6. Photochemical reactions are enhanced by the solar radiation to incur photochemical processes that generate aerosol precursors such as sulfuric acid and non-volatile vapors. These molecules can coagulate with each other to form a cluster leading to the NPF or condense upon pre-existing particle surfaces and then disappear in the sink process. Radiation also creates a turbulence flow that influences the nucleation regime by increasing supersaturation and accelerating the formation of clusters in the nanoscale. Enhanced turbulence could also dilute pre-existing particle concentration and enlarge the distance between the newly formed particles. The impact of turbulence on NPF may take place in two stages, strengthening the source in the nucleation process and reducing the sink in the growth process. As such, NPF could have a longer duration and higher growth rate (GR) as turbulence strengthens.

## CONCLUSION

NPF is a key process for haze formation, leading to air-quality deterioration. Chemical and photochemical processes have been intensively studied for understanding their roles in the NPF in the past decades, but the physical process has drawn much less attention. In this study, a ubiquitous relationship is found between the atmospheric stability intensity in the surface layer and the NPF features, based on a large number of observations in three sites in different countries (China, Finland and the USA). Numerous factors impacting the NPF are identified and quantified in our observation analyses and simulations by a molecular-dynamics model. Turbulence generates higher local supersaturation that facilitates condensable vapor to be clustered to form new particles and thus favors the nucleation process. Enhanced turbulence also dilutes the pre-existing particle concentration, causing the CS to decrease, favoring the growth of newly formed particles, which also prolongs the duration of NPF events. These findings suggest a new physical mechanism that may act on top of the traditional mechanisms of the NPF that are solely based on chemical and photochemical processes. It helps to elucidate the NPF process from a physical perspective that may improve the prediction of occurrence and duration of haze events.

## METHODS

### Definition of the Monin–Obukhov length

The Monin–Obukhov length (*L*) represents the turbulence stability, according to the Monin–Obukhov similarity theory. *L* is a parameter used to scale the turbulence stability in the surface layer, which accounts for both mechanically produced wind shear and thermodynamic gradient buoyancy, as well as unstable conditions [74,75]. *L* is expressed as

(1) \begin{equation*} L = \frac{{ - u_*^3{T_v}}}{{kg{Q_{vo}}}}, \end{equation*}

where u_*_ represents the friction velocity ($u_*\ = \sqrt { - \overline {u{\rm{^{\prime}}}w} {\rm{^{\prime}}}} \ $), *T_v_* the mean virtual potential temperature, *k* the von Kármán constant (∼0.41), *g* the gravity and ${Q_{vo}}$ the surface virtual potential temperature flux.

### CS calculation

The CS describes the loss rate (in molecules^−1^) of a vapor to an aerosol particle in the atmosphere. When the particle concentration is high, the sink becomes a limiting factor in the formation of new particles [76]. The CS [77] is calculated as

(2) \begin{eqnarray*} {\rm{CS}} &=& 2\pi D\mathop \int \limits_0^\infty {D_p}{\beta _M}\left( {{D_p}} \right)n\left( {{D_p}} \right)d{D_p} \\ &=& 2\pi D\mathop \sum \limits_i {\beta _M}{D_{f,i}}{N_i}, \end{eqnarray*}

where *D* represents the diffusion coefficient of the condensing vapor, which is usually assumed to be sulfuric acid (0.80 × 10^−5^ was used); *D_p_* represents the particle-size distribution; and *N_i_* represents the particle-number concentration. The term *β_M_* is defined as

(3) \begin{equation*} {\beta _M} = \frac{{1 + K_{n}}}{{1 + 1.677K_{n} + 1.333K_{n^2}}}, \end{equation*}

where *K_n_* = 2λ/dp. If  *K_n_* < 1, the droplet is said to be in the continuum regime and macroscopic laws, such as Fick's law of diffusion or Fourier's law of thermal conduction, can be applied. In the kinetic regime, i.e. *K_n_* > 1, kinetic gas theory can be used to calculate collisions and the resulting heat or matter exchanges between the particles and gas-phase molecules.

### Sulfuric-acid-concentration estimation

The UVB band (280–320 nm) solar radiation (UVB) and SO_2_ concentration (SO_2_) are used to estimate the proxy sulfuric acid (the sulfuric-acid concentration during the NPF window is shown in SI 2) by the following empirical equation [78]:

(4) \begin{equation*} \ \left[ {\rm{H_2}{\rm{S}}{\rm O_4}} \right] = {\rm{\ }}280.05 \cdot {\rm{UV}}{{\rm{B}}^{0.14}} \cdot {\left[ {{\rm{S}}{\rm O_2}} \right]^{0.40}}. \end{equation*}

### Coagulation-sink calculation

The coagulation sink (CoagS) is the governing component of NPF events because coagulation causes the loss of any new particles upon formation and thus shortens the event lifetime. The rate of coagulation depends on the pre-existing available surface area with which smaller particles can collide, which is derived from the aerosol general dynamic equation from discrete to continuous form:

(5) \begin{equation*} {\rm{CoagS}}{{\rm{\ }}_m} = \mathop \int \limits_0^{ + \infty } {\beta _{\left( {i,m} \right)}}\ {n_i}d{d_{i}}\!, \end{equation*}

where *β* is the Brownian coagulation coefficient among different sizes of *i* and *m, n_i_* is the total concentration and *d_i_* is a certain diameter. The CoagS provides the main loss mechanism, shortening the average lifetime [79].

### Geometric mean diameter (GMD) using log-fitting to determine the GR

The observed particle GR is calculated based on the lognormal distribution function method described by Kulmala *et al.* [80]. A typical particle-size distribution is fitted by a least-squares lognormal fitting method, yielding the parameters of one lognormal mode [81]. After the temporal variation in the GMD is obtained (Supplementary Fig. 15), the particle GR can be estimated (in units of nm h^−1^) [82]. The first step is fitting the GMD of the particles in the modal range during a specified period:

(6) \begin{equation*} {\rm {GMD}}\left( {{d_g}} \right) = {{\rm exp}}\frac{{\mathop \sum \nolimits_i \left( {ln{d_{\!P}}}^i \right)\ \times {N_i}}}{{\mathop \sum \nolimits_i {N_i}}}, \end{equation*}

(7) \begin{equation*} \rm {GR} = \frac{{\Delta \rm {GMD}}}{{\Delta t}}, \end{equation*}

where *d_g_* represents the GMD of the particles, *d_P_^i^* represents the particle diameter of bin *i* and *N_i_* represents the particle concentration in bin *i*. The observed particle GR is defined as the rate of change in diameter *d_P_^i^*, representing the growth of the particle population.

## DATA AVAILABILITY

The data and code used in the study are available from the authors upon request (wcgse@live.cn).

## Supplementary Material

nwaa157_Supplemental_File
