# Supplementary material for: The impact of the atmospheric turbulence-development tendency on new particle formation: a common finding on three continents
Source: Natl Sci Rev. 2020 Jul 6;8(3):nwaa157. doi: 10.1093/nsr/nwaa157 (PMC8288356; doi:10.1093/nsr/nwaa157)
Supplement: nwaa157_Supplemental_File [file nwaa157_supplemental_file.docx]

Supplementary Information for

**The impact of the atmospheric turbulence development tendency on new particle formation: a common finding on three continents**

Hao Wu^1^, Zhanqing Li^2,*^, Hanqing Li^3^, Kun Luo^3,*^, Yuying Wang^4^, Peng Yan^5^, Fei Hu^6^, Fang Zhang^1^, Yele Sun^6^, Dongjie Shang^7^, Chunsheng Liang^8^, Dongmei Zhang^1^, Jing Wei^1^, Tong Wu^1^, Xiaoai Jin^1^, Xinxin Fan^1^, Maureen Cribb^2^, Marc L. Fischer^9^, Markku Kulmala^10,11,12^, and Tuukka Petäjä^10,12^

*Correspondence*: Zhanqing Li ([zli@atmos.umd.edu](mailto:zli@atmos.umd.edu)), University of Maryland;

Kun Luo ([zjulk@zju.edu.cn](mailto:zjulk@zju.edu.cn)), Zhejiang University.

**This PDF file includes:**

Supplementary text

Figs. S1 to S15

Tables S1 to S4

References cited in supplementary text **1. Measurements**

**1.****1 Station locations**


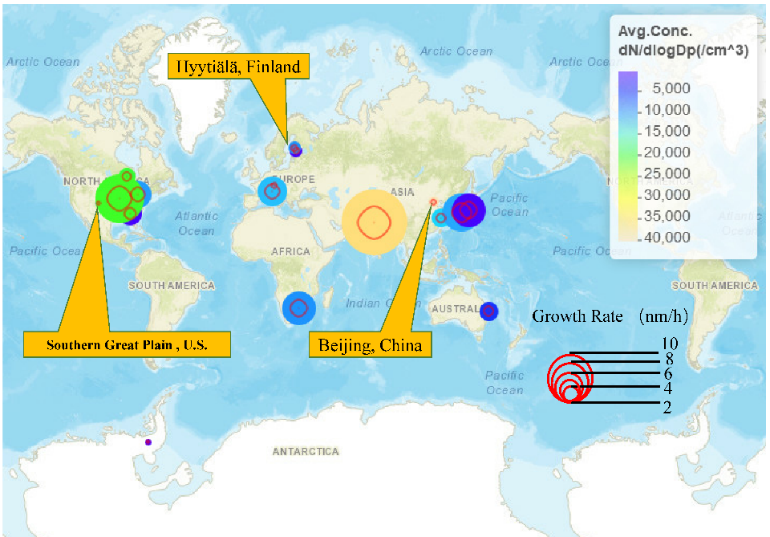


**Fig. S1. Sites locations and NPF features at each site. The fill color represents the average particle number concentration in that region. The size of the red circles shows the average growth rate** [1-15].

**1.1.2 Observations at the** **Southern Great Plains (SGP) site**

Using measurements from three SMPS instruments (DEG SMPS (1.9-13.9 nm) -NSMPS (2.8-47 nm) -RSMPS (23-528 nm), we got integrated particle size distributions from nominally 2 nm to 500 nm mobility diameter. Measurements were carried out every 5 minutes. Turbulence data (height at 4 m) were acquired in 2013 at the Atmospheric Radiation Measurement SGP field site in north-central Oklahoma (<https://www.arm.gov/data>), from which the impact of turbulence parameters on NPF events was verified. Merged data from the scanning mobility particle spectrometer (SMPS) using diethylene glycol as the working fluid and data from the nano SMPS and regular SMPS, omitting the first 14 channels of the SMPS (starting at ~14 nm). The growth rate and duration of each NPF event were determined in the same way as was done for Beijing [16, 17]. The lower limit of PNSD data was sub-3 nm, and eddy covariance data at the 4 m level were collected to retrieve the stability parameter ζ. Unified the timestamps of all data files, some of which were in UT and others in Central Standard Time. growth by primary organics, primarily sulfuric acid and ammonia are researched by Hodshire [18].

**1.1.3. Long-term observations at the SMEAR** II **/HYY forest site**

Hyytiälä (HYY) is a long-term observatory built by Helsinki University that belongs to the network of the Station for Measuring Forest Ecosystem-Atmosphere Relations (SMEAR II) in Finland (https://avaa.tdata.fi/). The HYY station is located in southern Finland (61.85^o^N, 24.28^o^E; 181 m above sea level), approximately 220 km northwest of Helsinki. Particle size distributions have been measured at the site for almost 24 years, starting in 1996[19, 20], with maximum total HOM concentrations in the spring [21]. The concentrations of HOM are more than one order of magnitude larger than those of sulfuric acid [22]. These observations imply that oxidized organics especially LVOCs and SVOCs [23] are related to the NPF [24]. Downloaded turbulence (height at 3 m) and particle size distribution data from 2013 to 2017 in a search for NPF events. Almost daily NPF events occurred from 8 March to 13 April 2013, so this period was chosen. We calculated the condensation sink and marked the results along with the turbulence fluctuation. The lower detection limit is 3 nm, which allows for studying the evolution of turbulence and the nucleation process.

**1.1.4 Description of the Beijing sampling site**


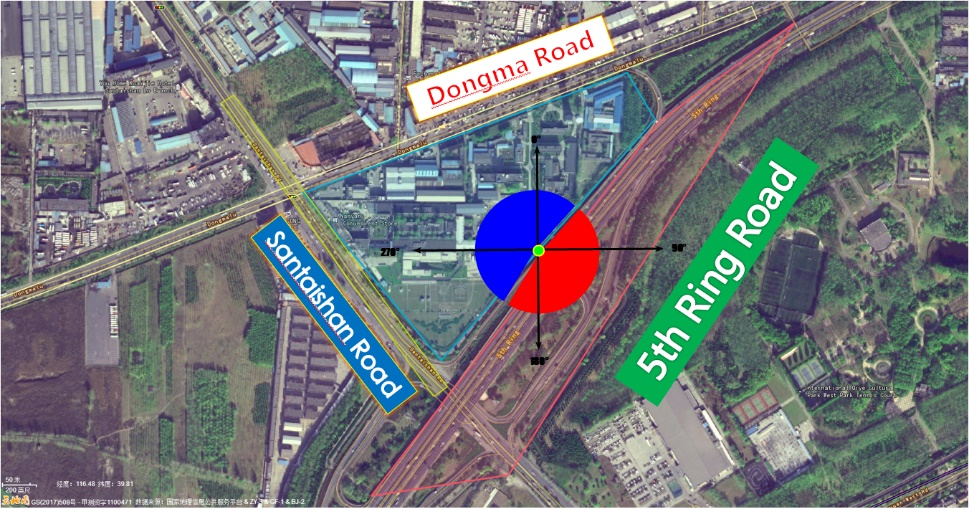


**Sample Site**

**Fig. S2. Map view of the sampling location.**

- 1. **Instrumentation**

The sampling site (39.80^o^E, 116.82^o^N) is located at the Beijing Metrological Observatory, which is surrounded by a highway (south 5th Ring Road, traffic flow: 183,186 cars/day on average; <http://www.bjtrc.org.cn/List/index/cid/7.html>). A forested park located to the southeast and two city roads (Santaishan Rd. and Dongma Rd.; traffic flow: 17,200 cars/day on average) surround the sampling site, and a residential region is located to the north. No significant local industrial emissions are produced nearby. This site is a representative site with a mixed source influence that includes traffic emissions, biomass burning, biogenic emissions and H_2_SO_4_[10, 25], and HOMs [26] from human activities [27, 28] and organics [29], other acid [30]. Traffic emissions are the predominant source of atmospheric aerosol particles [31]. This site provides a unique opportunity to estimate the contribution of traffic emissions to NPF occurrences and to understand the correlation between such emissions at the ground level. This would help improve model simulations because the impact of exhaust emissions is always underestimated.

A container on a grassy surface housed the measurement system. Aerosol samples were aspirated at the rooftop (~4 m above the ground) through a cyclone inlet (2.5-µm cutoff size) connected to stainless steel tubing with an internal diameter of 6 mm, and with a flow stay length of less than 4 m. All sample inlets were lower than the road subgrade so that [vehicle](file:///C:\Users\WuHao\AppData\Local\youdao\dict\Application\7.5.2.0\resultui\dict\?keyword=vehicles) dust and other gas exhaust could directly reach the sample inlet tube. The system airflow was dried through a tube containing silica gel, and the infill material was changed approximately twice a month to ensure that the relative humidity was always below 30%. The refill frequency was dependent on environmental conditions. Table S1 provides an overview of all systems deployed for field observations. All systems were on-line and operational under all-weather conditions. Despite calibration errors and malfunctions caused by hardware or software problems, sufficient data were collected to study NPF events. All instruments were synchronized to the same timestamp.

**1.2.1. Particle number size distribution**

The SMPS model 3938 system utilized in the study was calibrated by polystyrene latex particles (40/80/100/200/400 nm) before and after each observation, with a bias of less than 5%. Compared were measurements made by condensation particle counter (CPC) model 3772 and a second standard CPC before each observation. The bias was within 10%. The system sample flow rate was set to 1.02 L/min, the sheath flow rate was maintained at 4.5 L/min, and the measured range of particle sizes was 11.3 to 552.3 nm, with 64 channels. The first data assurance step was the multiple charge correction, implemented because particles with multiple charges have increased electrical mobility, causing them to be counted in a larger size bin. This step is followed by the diffusion loss correction, implemented because particles with small diameters (<100 nm) can easily diffuse to the tube walls of the SMPS spectrometer aerosol flow path and get lost [32]. The third step is restricting the detector counting efficiency to 20%, implemented because near the small end of their detectable range, particle detectors, such as CPCs, have reduced counting efficiencies. Large corrections caused by a low counting efficiency were prevented by default because the counting efficiency correction was restricted to a minimum value of 20% (the maximum correction factor set to 5).

- - 1. **Eddy covariance flux and meteorological measurements**

The Li-COR 7500A system measured the thermodynamic quantities of static air pressure, air temperature, and water vapor density, and the 3-D wind vector was applied for meteorological measurements of wind and turbulent fluctuations. The time resolution was averaged to 5/10 min. Fluxes of CO_2_/H_2_O, sensible heat, latent heat, and momentum were measured at a height of 2.1 m from the ground with an open-path eddy covariance system and recorded by a CR5000 data logger (model CR5000, Campbell Scientific, Logan, UT, USA). Flux data were analyzed in half-hour intervals [33]. Additional meteorological parameters, such as global solar radiation, net radiation, and photosynthetic photon flux density, were observed at a height of 1.8 m above the ground. Data were processed using the Eddy Pro procedure (Li-COR, version 6.2.1) then output to Tovi (Li-COR, version 2.2.1), which performs active database quality control and variable merging [34].

**Table S1. List of instruments and parameters.**

|  | Instrument | Parameter | Manufacturer | Model | Time Resolution |
| --- | --- | --- | --- | --- | --- |
| **Aerosol**  **Properties** | SMPS | Particle number size distribution | TSI | 3938 | 5 min |
|  | Neutralizer | Particle changing | TSI  85Kr | 3088 | 5 min |
|  | DMA | Particle classifier | TSI | 3081 | 5 min |
|  | CPC | Condensation particle counter | TSI | 3787/3772 | 5 min |
|  | APS | Aerodyne size distribution | TSI | 3321 | 5 min |
|  | CCNc | Cloud condensation nuclei counter | DMT | 100 | 15 min |
|  | ACSM | Aerosol composition | Aerodyne | Q- ACSM | 15 min |
| **Solar**  **Radiation** | Pyranometer | Net irradiance | Kipp & Zonen | CNR4 | 1 min |
|  | PAR | Photosynthetically active radiation | Li-COR | LI-190R | 1 min |
| **Gas Analyzers** | Non-dispersive infrared | CO_2_ concentration | LI‑COR | Li-7500A | 5 min |
|  | Gaseous precursors | SO_2_ /NO_x_ concentration | Thermo Fisher | 43i/42i | 5 min |
| **Meteorological**  **Parameters** | Sonic anemometer | Sonic wind | Gill | Wind Master Pro | 1 s |
|  | T/RH | Temperature/relative humidity | Vaisala | HMP 155A | 1 s |
|  | SmartFlux2 | Data logger | LI‑COR | Smart Flux2 | 1 s |

**1.3 Wind direction and wind speed influence**

During the observation period, the wind direction can alter the particle size distribution through different air masses from the direction of the particle source. However, distinguishing noise from the signal is a challenge in the process of the lognormal fitting method. Noise is difficult to separate from the fitted geometric mean diameter because background noise during a specified research period is positively correlated with wind direction, which can be thought of as a local traffic emission source. However, turbulence-induced air transport should be considered when NPF happens in the vertical wind shear mixing. A case in Beijing on 7 August 2017 illustrates this (Fig. S3). The wind direction suddenly changed from northerly at ~06:00 local time (LT) to southerly at 07:00 LT (wind direction around 180°), resulting in a peak in the particle distribution. This shows that pollution from the south can be transported to the site. Around 09:00 LT, the predominant wind was from the northeast, i.e., where clean air masses originate. Local background particles were dispersed, creating pristine circumstances favorable to NPF. At approximately 15:00 LT, the wind direction shifted from east to west, and the nucleation mode (less than 25 nm) showed significant background noise (wind direction around 200°) below 50 nm. When the wind changed to the west (wind direction around 270°) at ~16:00 LT, traffic sources persisted until NPF ended at approximately 12:00 LT on 8 August. In this case, the change in wind direction had no significant impact on the NPF growth process itself.


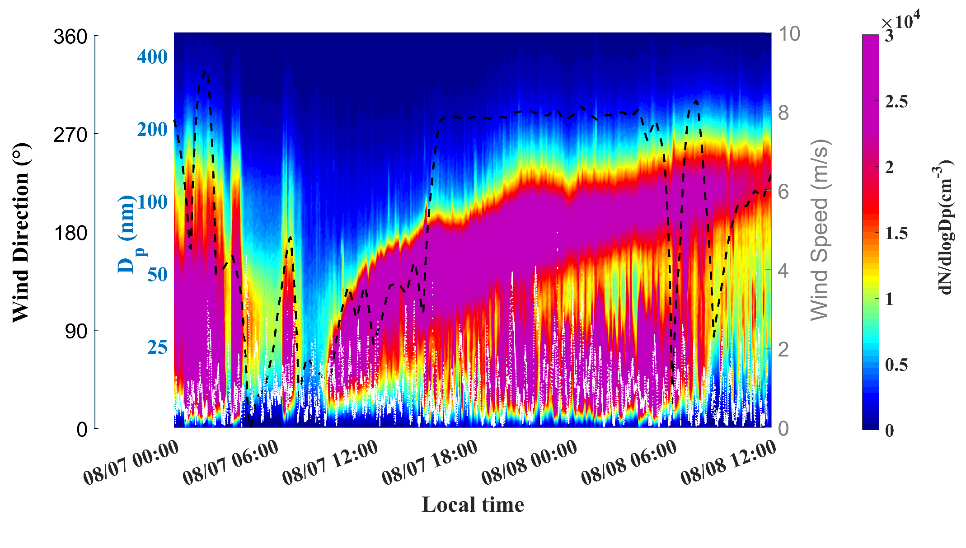


**Fig. S3. Influence of wind speed and direction on the particle number size distribution. Wind direction is indicated by the black dashed line, wind speed is indicated by the white line, the y-axis indicates the particle diameter (Dp), and the color map represents the number concentration.**

Quantification analyses of these cases help further understand the influence of turbulence parameters on NPF events. A total of seven NPF events occurred from 12 March to 1 April 2019 (Fig. S4) based on criteria established by Kulmala [35]. First, the nucleation mode number concentration shows a clear burst in a short time, persisting in the atmosphere for a few hours and then subsequently growing to an accumulation mode or even larger, with an event duration time exceeding two hours. The stability parameter **ζ** sharply drops before the beginning of an NPF event. Next, calculated is the total duration time of the sustained increase from nucleation in all cases, and investigated is the type of atmospheric stability condition that may favor NPF events.


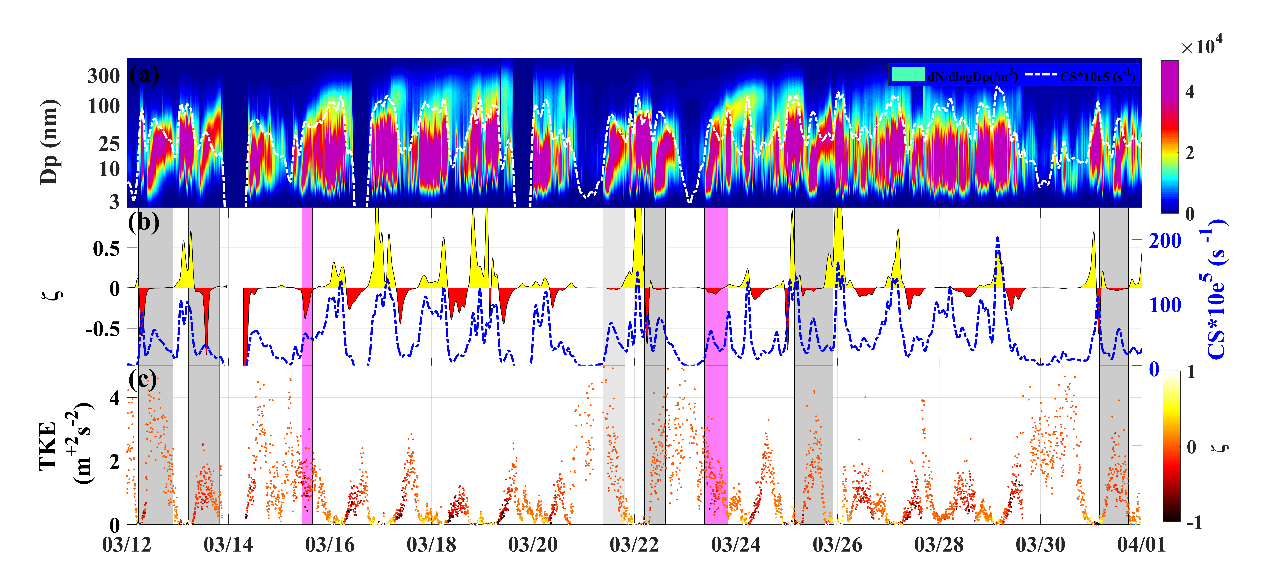


**Fig. S4. (a) Evolution of the particle number size distribution (legend on the right; the white dashed line is the condensation sink, CS*10^5^) at the Beijing site from 12 March to 1 April 2019. (b) Evolution of ζ. The red and yellow areas show turbulence development during unstable and stable conditions, respectively. (c) Evolution of turbulent kinetic energy filled with ζ (TKE left ordinate and ζ right ordinate). The grey- and magenta-colored areas show different cases, with their widths representing the duration of each NPF event.**

To investigate how atmospheric stability affects NPF events, two stability conditions are defined: a stable condition (stable atmospheric stability when **ζ** > 0) and an unstable condition (unstable periods occurring when **ζ** < 0, increasing with | **ζ** |). Once the surface layer transitions to an unstable regime, the condensation sink (white dashed line in Fig. S4a) shows a synchronal decreasing trend. A burst in the nucleation mode concentration then occurs, leading to the growth of newly nucleated particles and causing the NPF “banana shape” to appear in the particle size distribution over time. These changes imply that a strong unstable condition can impact the gas-solid phase transformation process.

**1.4. Correlations between NPF and related parameters**


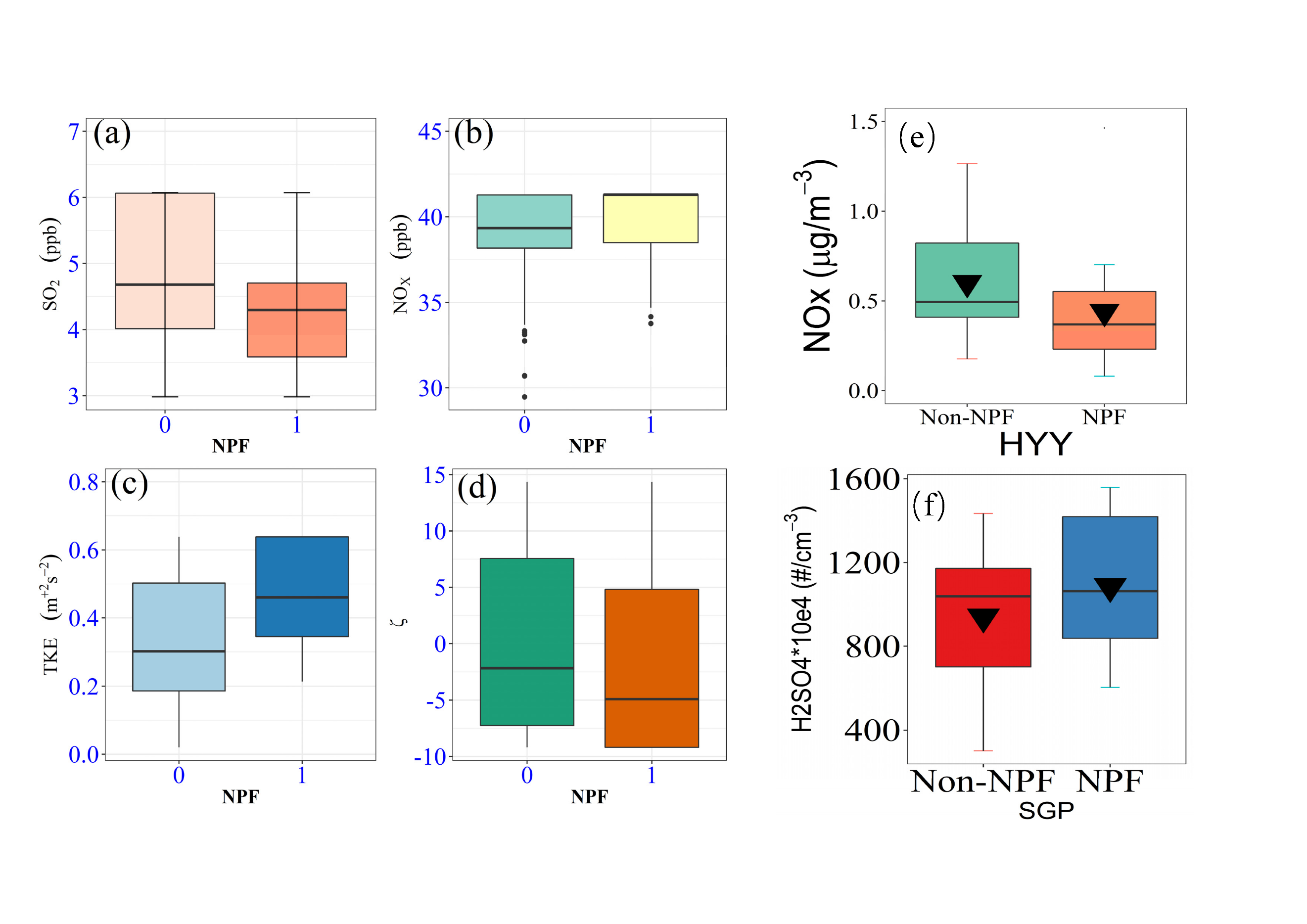


**Fig. S5. Comparisons between NPF and related parameters** **in Beijing. Boxplots of selected parameters: (a) SO_2_ concentration, (b)** **NO_x_ concentration, (c) turbulent kinetic energy (TKE), and (d) the ζ during NPF days (1 on the x-axes) and non-NPF days (0 on the x-axes). (e) NO_x_ concentration at HYY, (f) H_2_SO_4_ concentration at SGP.**

Gaseous precursors of SO_2_ are lower on NPF days (Fig. S5a), and the concentration of NO_x_ is higher on NPF days (Fig. S5b), compared with non-event days. However, the TKE and **ζ** (negative **ζ** values are positively correlated with the turbulence intensity according to the definition of **ζ**) differ on NPF days (Fig. S5c, d). TKE is much higher, and **ζ** is lower on an NPF day than on a non-NPF day. This suggests that the turbulence is stronger on NPF days. NPF occurrence is highly related to higher NO_x_ concentrations but lower SO_2_ concentrations, as well as higher TKE and lower **ζ**. All of these variables are highly correlated with solar radiation. Fig. S5e NOx concentration at HYY, Fig. S5f H_2_SO_4_ concentration at SGP are provided to further compare the difference between NPF event days and Non-NPF event days, H_2_SO_4_ concentration is much higher in SGP, but NOx concentration obviously lower than NPF days in HYY.


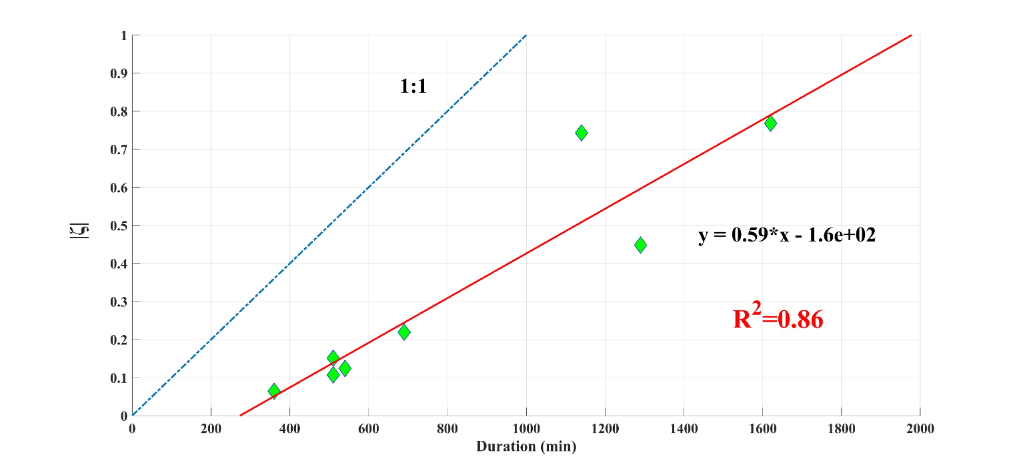


**Fig. S6. Correlation between ζ and NPF duration.**

A positive correlation is observed between the strength of instability intensity |ζ| and the duration of each NPF event, with more atmospheric instability leading to longer durations (Fig. S6). The correlation between ζ and duration reaches 0.86. One possible explanation for this relationship is that unstable surface atmospheric space generates more local supersaturation, which is essential in the first step of nucleation, fostering more condensation gas to participate in the transformation of the gas phase to freshly formed particles.

**2. NPF window average parameter evolution**


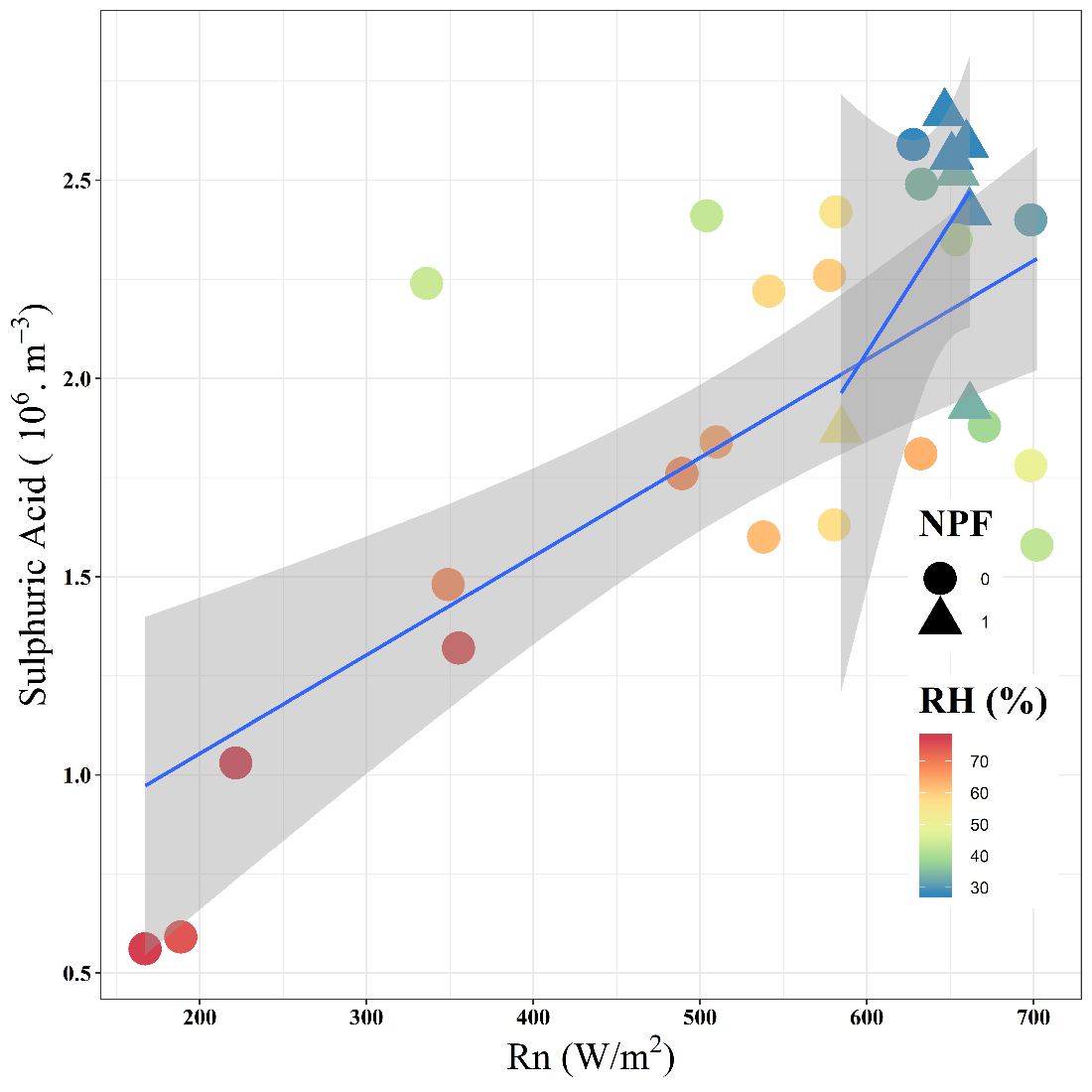


**Fig. S7. Correlation between** **sulfuric acid concentration and net radiation (R_n_) where the color of the symbols indicates the relative humidity level. NPF event days are labelled as 1 (triangles) and non-event days are labelled as 0 (circles).**

Calculated the average values in the NPF window period (09:00–15:00 LT) to better understand the association between key parameters and NPF occurrence. Fig. S7 shows that more intense solar radiation creates more sulfuric acid, and low relative humidity levels promote the production of sulfuric acid, especially on NPF days. The concentration of sulfuric acid reached a peak value of ~2.5x10^6^ #/cm^3^, partly explaining the NPF event. However, the H_2_SO_4_ concentration alone is not positively correlated with NPF events because the sulfuric acid level is much higher on some non-NPF days. The association between the condensation sink and ζ should thus be further investigated.

**
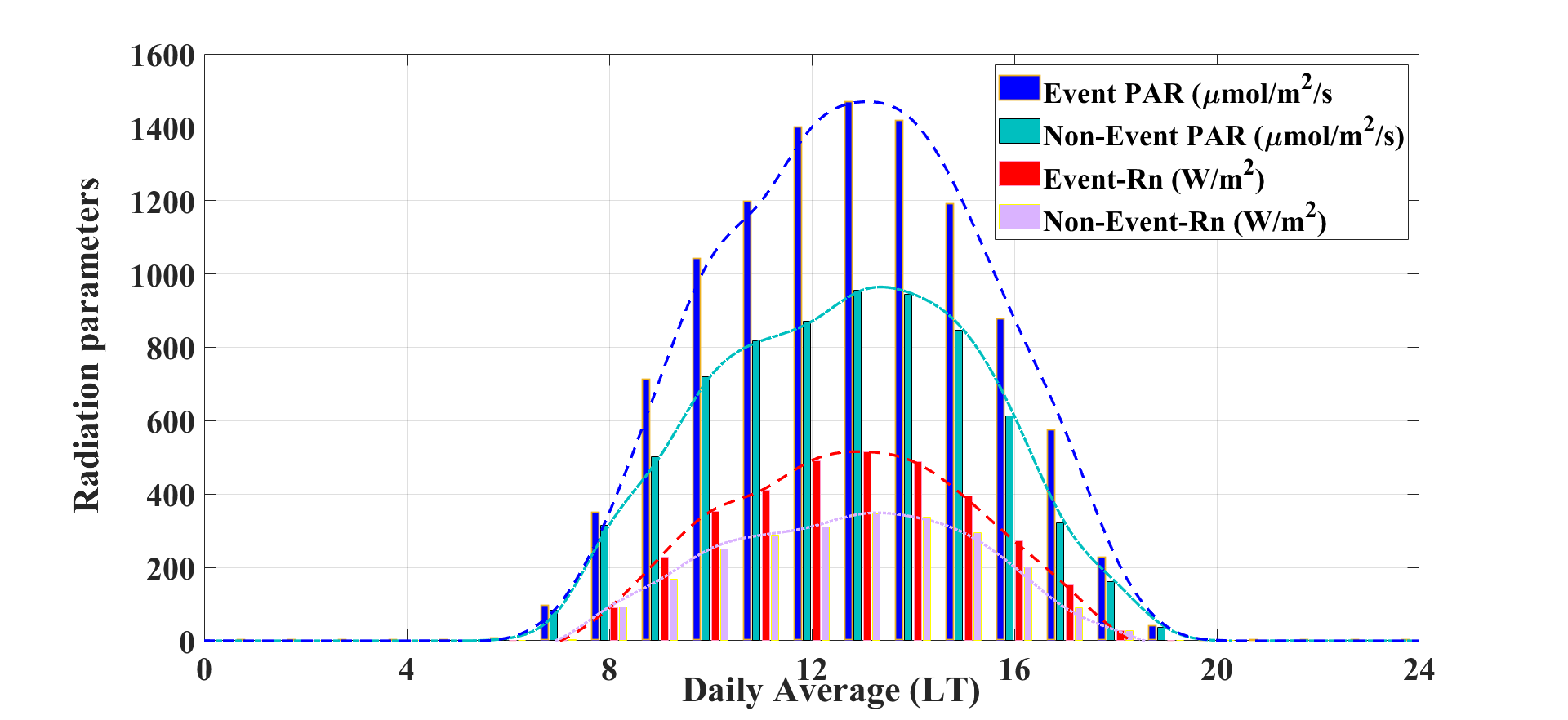
**

**Fig. S8. Photosynthetically active radiation (PAR) and net radiation (R_n_) on different event days.**

Photosynthetically active radiation or photosynthetic photon flux density (μmol photons m^−2.^s^−1^) can reflect the intensity of the change in bandwidth and is more suitable for energy balance studies. Incoming radiation produces OH radicals, which act as another surrogate for photochemical production. Particle nucleation processes include the formation of sulfuric acid, which is also linked to OH radical production. In some cases, water vapor affects nucleation through its influence on OH radical production via ozone photolysis. An unstable atmosphere causes surface turbulence fluctuation, which can create an initial burst of nanoparticles [36]. Higher radiation levels increase turbulence and generate more local supersaturation, activating a regime that tends to let all gases that are clustering become condensation sources [37]. All these factors are integrated into the first step of nucleation, and highly unstable flows induce more gaseous condensation in the transformation of the gas phase into newly formed particles. In general, in a physical regime with no barriers, two molecules from different sources can meet to form a dimer, and a two-molecule cluster may evaporate back to its monomers at a lower rate, or collide with a third molecule to form a trimer and then a cluster. This physical process could be easily influenced by atmospheric stability. For example, turbulence may induce eddies in the fluid flow, and then clusters will be compressed and can break up the kinetic energy barrier to form a particle. Due to turbulent diffusion, enhanced turbulence can dilute preexisting particles effectively, which also exerts an influence on the particle size distribution and can favor the growth of freshly nucleated particles [38]. Clusters and more particles are formed when supersaturated vapor in the system is governed by turbulent flow.

**3. Case study**

**3.1 Case studies of the impact of atmospheric stability on NPF events in Beijing**


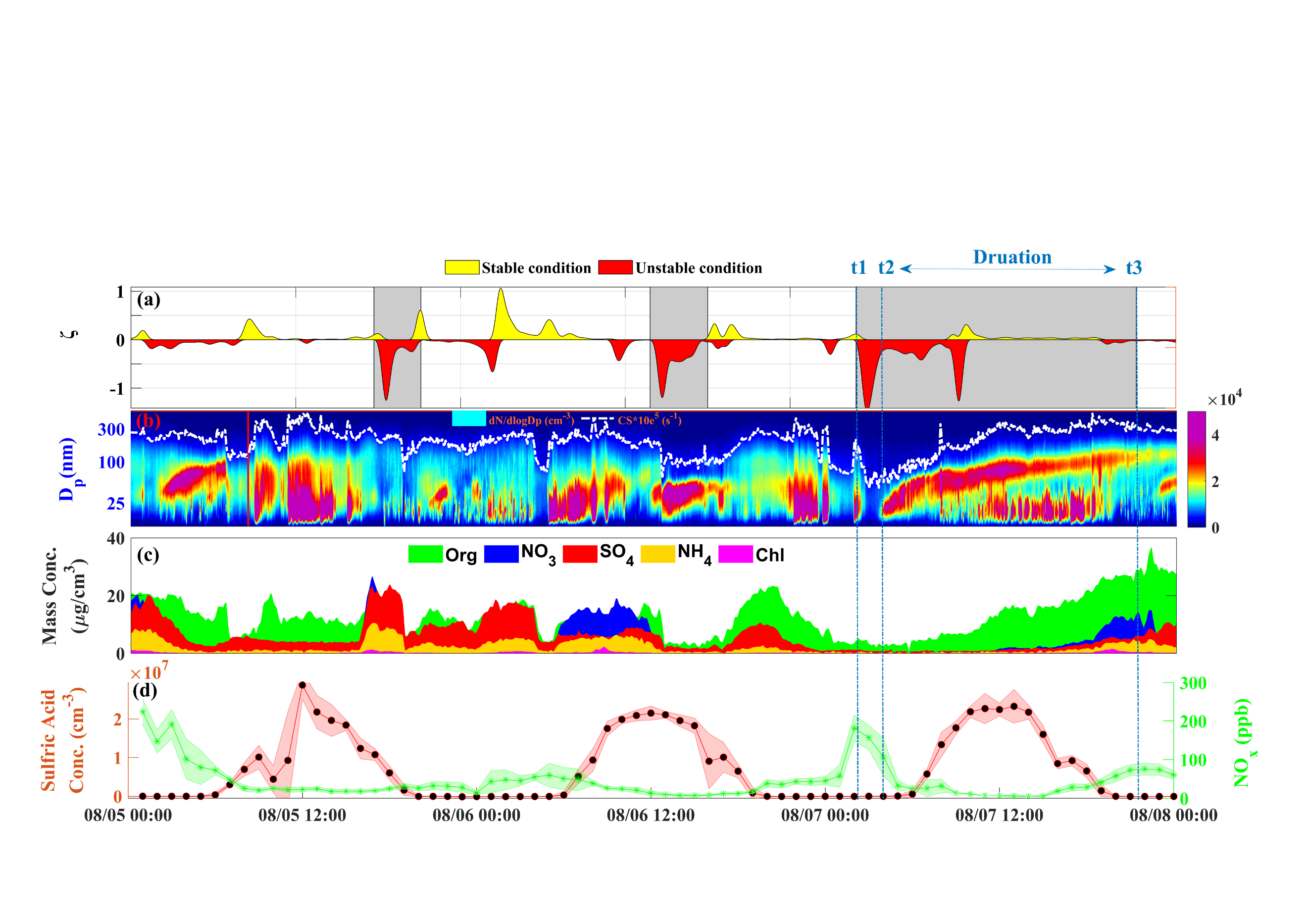


**Fig. S9. The time series of summer NPF events and associated variables in Beijing: (a) stability parameter ζ, (b) particle number size distribution (CS*10^5^, indicated by the dashed white line), (c) aerosol mass concentrations of chemical species, and (d) estimated sulfuric acid (left ordinate) and NO_x_ (right ordinate) concentrations, with the blue dashed lines marking t1 to t2 to indicate the period of 2 h prior to NPF and t2 to t3 defined as the duration of NPF.**

Fig. S9 illustrates the atmospheric stability, showing a significant downward fluctuation before the NPF event compared to that on the non-event day, indicating that turbulence has some association with the occurrence of an NPF. In Fig. S9a, negative values of ζ indicate that the atmosphere near the surface became unstable during the period prior to an NPF (t1 to t2). A classic NPF event occurred at approximately 08:30 LT on 7 August 2017 (t2 in Fig. S9b) and then grew into the accumulation mode (100 nm), ending near 23:00 on 7 August 2017 (t3 in Fig. S9b). The mass concentration of organics (green area in Fig. S9c) grew sharply after the NPF burst. The estimated sulfuric acid concentration had no significant variability between these days. However, the diurnal variations in NOx concentration peaked at night and gradually decreased after sunrise, likely an indicator of an NPF (Fig. S9d). our results revealed that preexisting particles provide surface area to foster the coagulation of nanoparticles, suppressing the occurrence of NPF. The evolution of the turbulence tendency is highly associated with most NPF events. A downward trend in ζ can be a proxy for increasing turbulence, of which the duration is represented by the length of the shaded areas (Fig. S9, t2-t3). Many NPFs occurred in the presence of both strong turbulence and a lower CS. Enhanced turbulence can dilute the preexisting particle concentration and lessen CS, thus favoring the formation of new particles. The turbulent stability intensity reached a high level before the start of an NPF event (Fig. S9a, gray shaded areas). The average ζ dropped significantly two hours before the particle bursts. Tables S2 to S4 summarize the other cases in Beijing and at other sites.


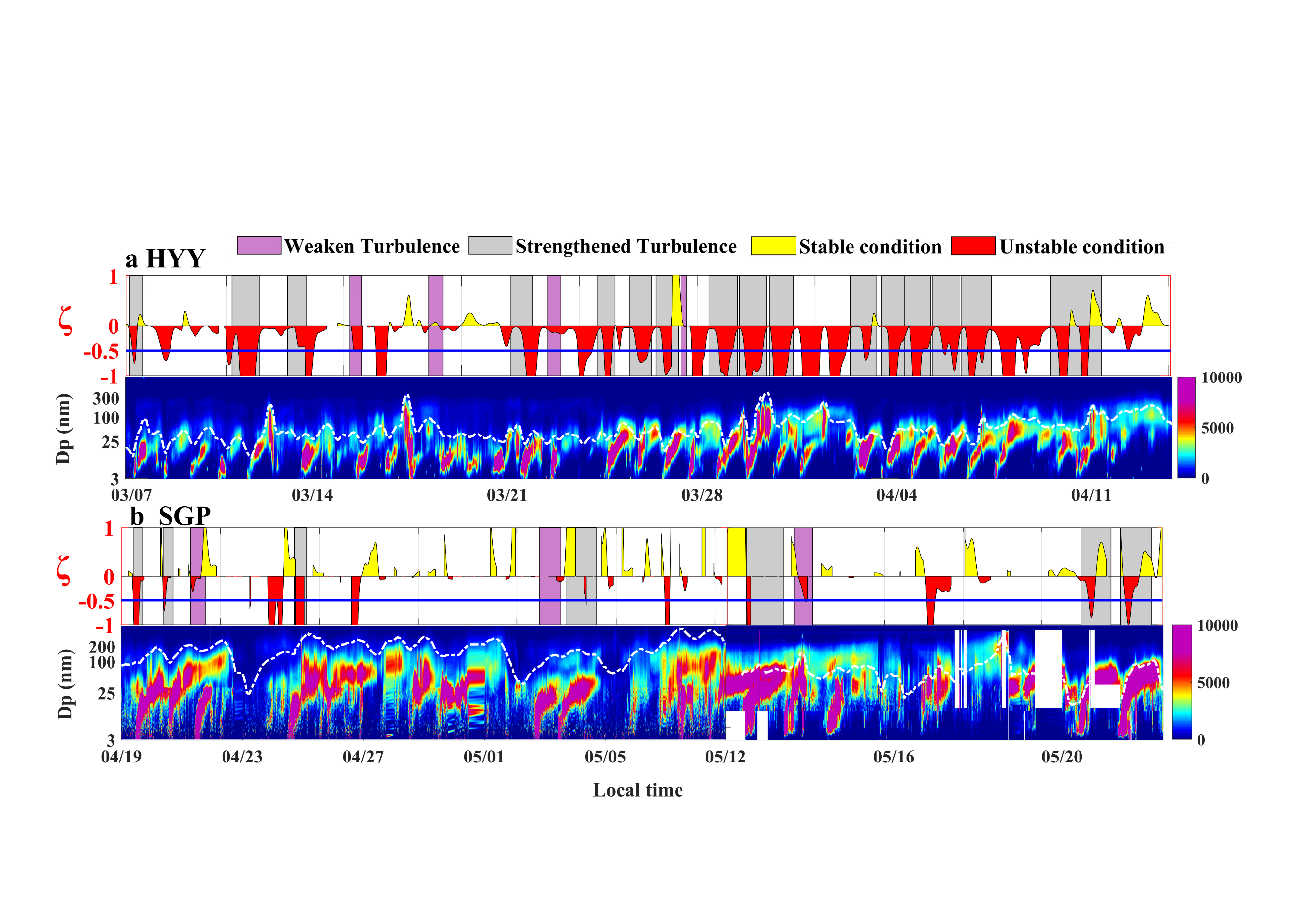


**Fig. S10. Evolution of ζ (upper figure of each subpanel) and the particle number size distribution (lower figure of each subpanel). The white dashed lines are the condensation sinks (CS*10^5^) at (a) the Hyytiälä (HYY) site in Finland and (b) the U.S. Southern Great Plains (SGP) site.**

Fig. S10 presents some examples of NPFs observed at the Hyytiälä (HYY), Finland, and at the U.S. Southern Great Plains (SGP) site. NPF events occurred almost every day from 8 March to 13 April 2013 at the HYY site. The CSs and turbulence fluctuations had the similar correlation. There were 11 NPF events in total at the SGP site, the size distribution measured by the scanning mobility particle sizer ranged from 3.0 to 521 nm from 19 April to 12 May and from 1.59 to 488 nm from 12–22 May. Although the chemical characteristics of the atmospheres at these sites are likely quite different from those at the Beijing station, a common feature is the turbulence stability in the surface layer, impacting the particle evolution as CS increases.

**3.2. Variations in the condensation sink with atmospheric stability parameters**


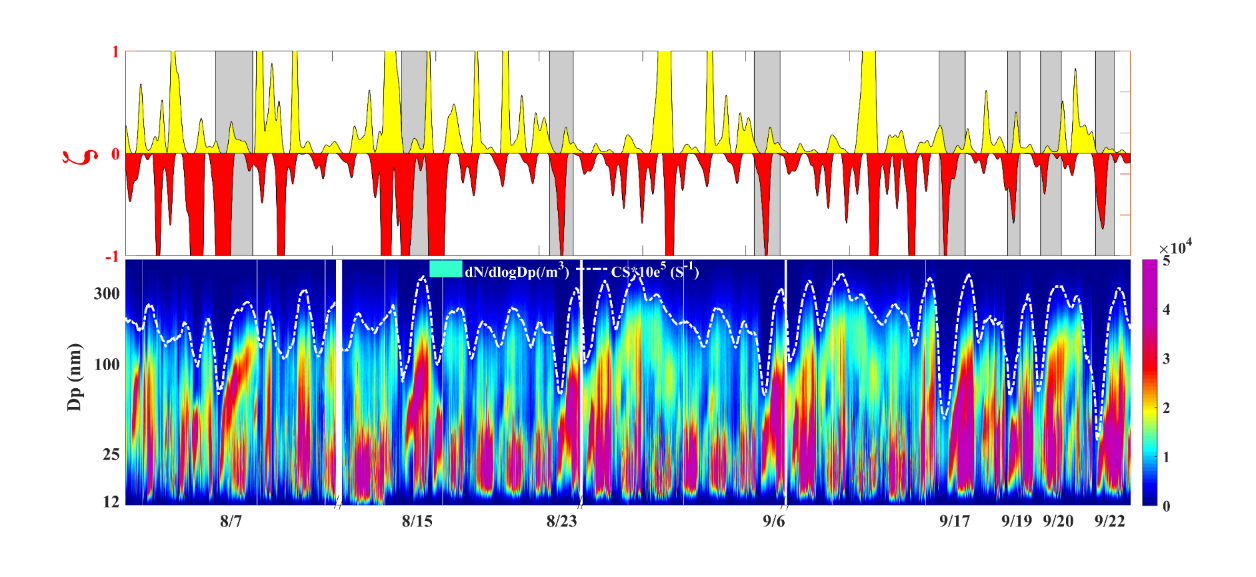


**Fig. S11. Variations of ζ (uppermost subpanel) with the particle number size distribution (lowermost subpanel, legend on the right), and the condensation sink (white dashed line, CS*10^5^) at the Beijing site from 7 August to 22 September 2017. In the uppermost panel, yellow areas denote stable periods, and red areas denote unstable periods.**

In the atmosphere, a large preexisting particle surface area can act as the host of the condensation sink, which is expected to decrease nucleation rates because the newly formed particles live for a very short time, neither growing via condensation nor disappearing via coagulation. Fig. S11 shows the evolution of the condensation sink during each NPF event. The atmospheric stability intensity becomes high instability before the burst starts (red areas in the uppermost panel of Fig. S11), accompanied by a decreasing condensation sink to its lowest point facilitating nucleation. This implies that an unstable surface can create a free space to dilute the preexisting particle concentration. larger clusters and small nanoparticles formed during a nucleation event need to grow quickly so that they are not scavenged by coagulation through collisions with larger particles. In this case, more clusters can participate in the growing process spontaneously, becoming an abundant particle source for a longer NPF event.

NPF events are easily observed when the background is pristine, and turbulence activates a regime that tends to allow all gases to transform into clusters and become a self-coagulation source, while also reducing the condensation sink. An unstable atmosphere enhances deposition, especially as the interception sink strengthens, and blockage of coagulation of small particles near the pre-existing particle surface occurs. This finding is similar to the likelihood that turbulent mixing will lead to the local supersaturation of possible precursor gases, which are essential for NPF. Dilution can effectively decrease the condensation sink, although coagulation and deposition sinks cannot be neglected in the NPF process. However, there are some exceptions. NPF does not happen when the condensation sinks are at a low level, or the atmosphere is unstable. A possible explanation is that accumulation-mode particles may have some feedback to intermittent turbulence [39].

**4.1 Condensation sink correlations with four concentration modes**


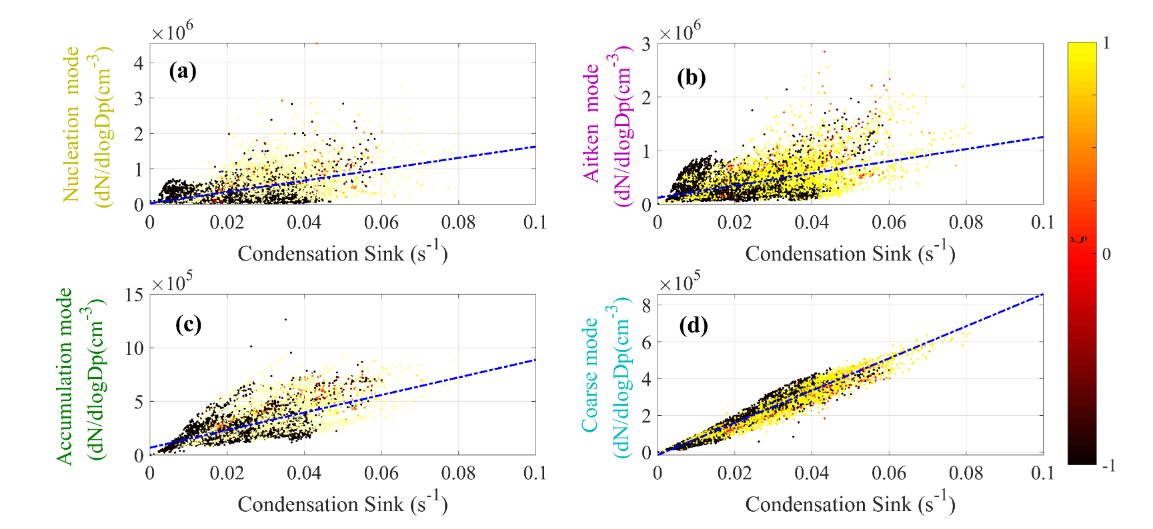


**Fig. S12. Four concentration modes as a function of the condensation** **sink: (a) nucleation mode (10.7–25 nm), (b) Aitken mode (25–100 nm), (c) accumulation mode (> 100 nm), and (d) coarse mode (> 1000 nm). The colored dots show the magnitudes of ζ (legend on the right). Dashed blue lines are the linear best-fit lines through the points.**

Particle concentrations can be divided into four modes, i.e., nucleation, Aitken, accumulation, and coarse modes. Fig. S12 shows particle concentrations of each mode as a function of the condensation sink and **ζ**. **ζ** values appearing when CS is less than 0.04 in all modes indicates that strong turbulence decreases the concentration of preexisting particles and helps reduce the CS. Large negative **ζ** values, which are almost always observed when CS levels are low, suggest that **ζ** has a significant effect on the concentrations of all modes. In addition, the coarse mode is the most sensitive to **ζ** fluctuations because turbulence can alter concentrations more substantially in this mode, compared with the other modes. Moreover, the CS level is dependent on the larger mode particle concentration and also affected by greater instability, suggesting that turbulence has a significant impact on the evolution of CS evolution.

**4.1.1 Other parameters influencing the condensation sink (CS) during the NPF window**

Fig. S13a shows that CS increased with increasing temperature filled with low RH on NPF days. The large aerosol particles due to hygroscopic growth provide surfaces upon which gas vapors can condense. This indicates that temperature and RH strongly influence CS. An increasing ambient temperature could cause semi-volatile organic species to be partitioned into the gas-particle phase. Temperature gradients caused by a phase transition due to the latent heat released or absorbed during the transition process may also affect CS. In Fig. S13b, the CS level decreased sharply when the atmosphere moved toward an unstable condition (i.e., increased |ζ|), with almost every NPF day showing the lowest CS level of ~0.01 s^-1^, accompanied by low SO_2_ concentrations (< 4 μg m^-3^). These observations suggest that higher instability help decrease the CS favor the NPF occurrence.


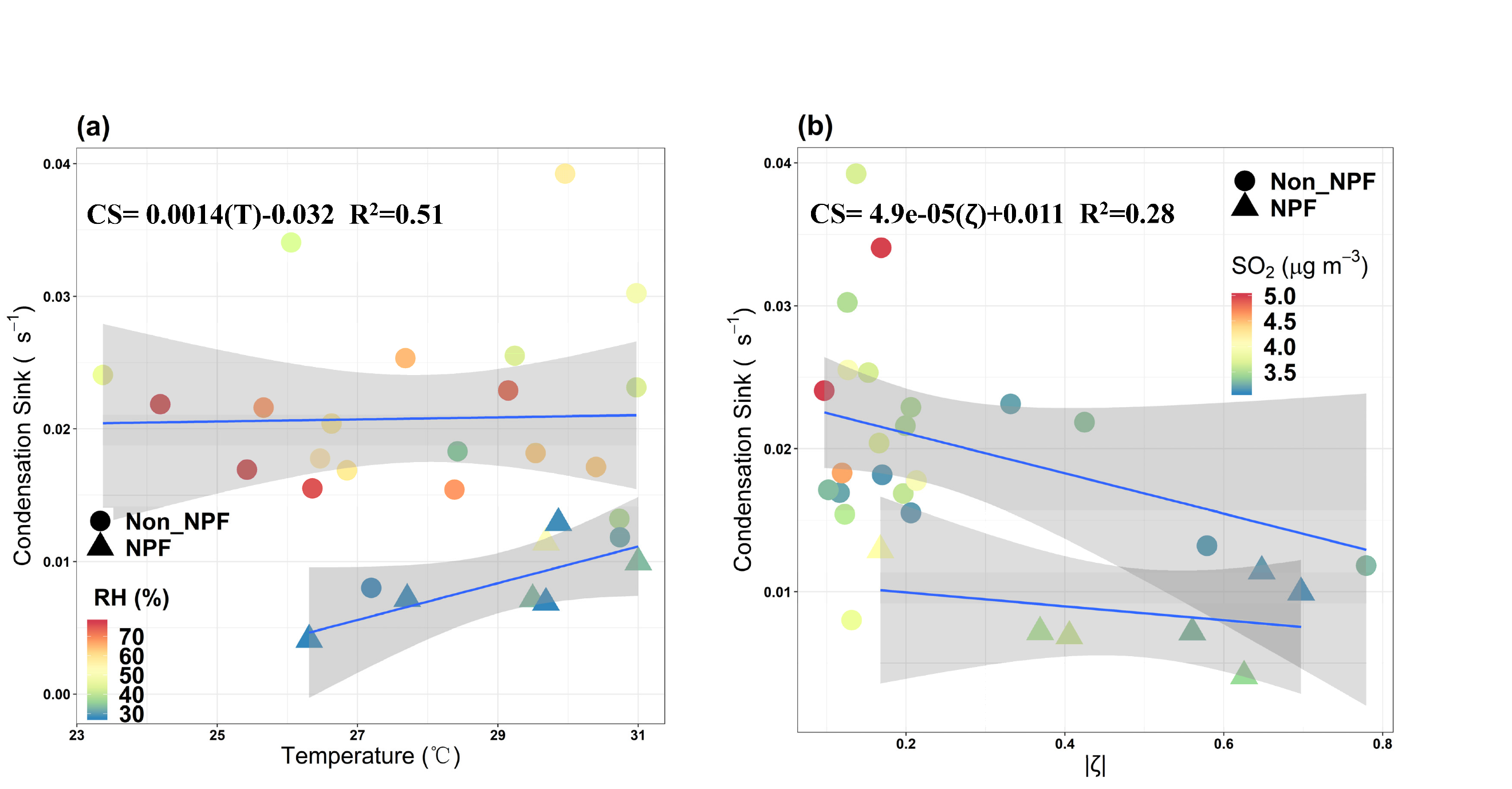


**Fig. S13. (a) Relationship between the condensation sink (CS) and temperature in Beijing, with colors indicating different RH values, and (b) relationship between the CS and |ζ|, with colors indicating different SO_2_ values. NPF event days are labelled as triangles, and non-event days are labeled as circles.**


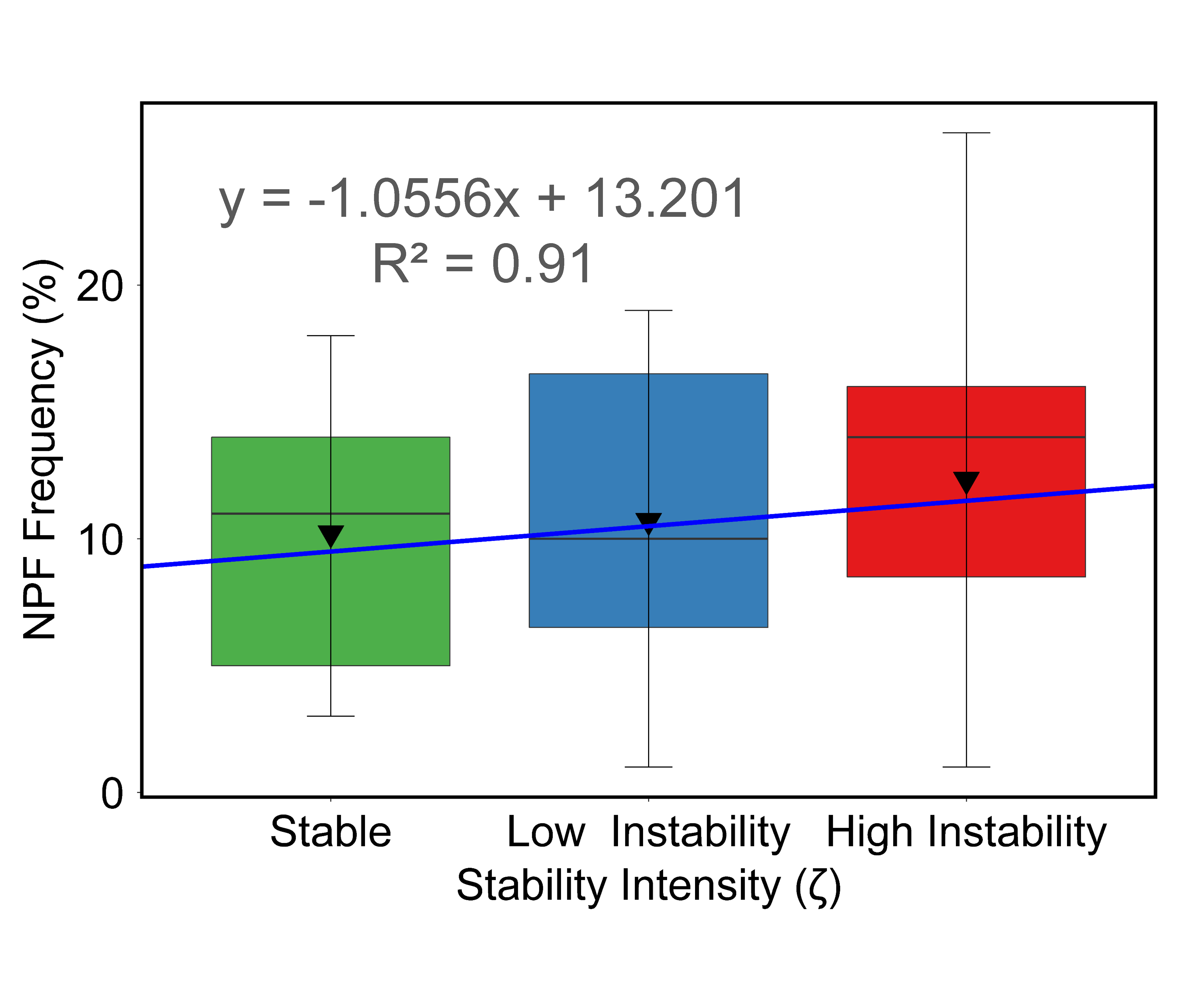


**Fig. S14. NPF Frequency in three stability intensity bins at Beijing with ζ > 0 is stable, low instability** [**percentile**](javascript:;) **is the -0.5<ζ<0, high instability percentile is the ζ<-0.5, boxes showing the 25th, 50th and 75th percentile, the extremities show the 5th and 95th percentiles.**

Fig. S14 shows NPF frequency at Beijing during 2017/7/19-2019/10/23, including 449 days after quality-controls. As the atmosphere changes from stable to low and high instability conditions, the NPF occurs more frequently, the correlation R^2^ is 0.91 after stability intensity was binned into three quantiles in our database. It suggests that more gas precursors are transformed into clusters and then grow into particles under strong turbulence conditions which causes more frequent occurrence of NPF.

**4.1.2. Geometric mean distribution analyses**

**
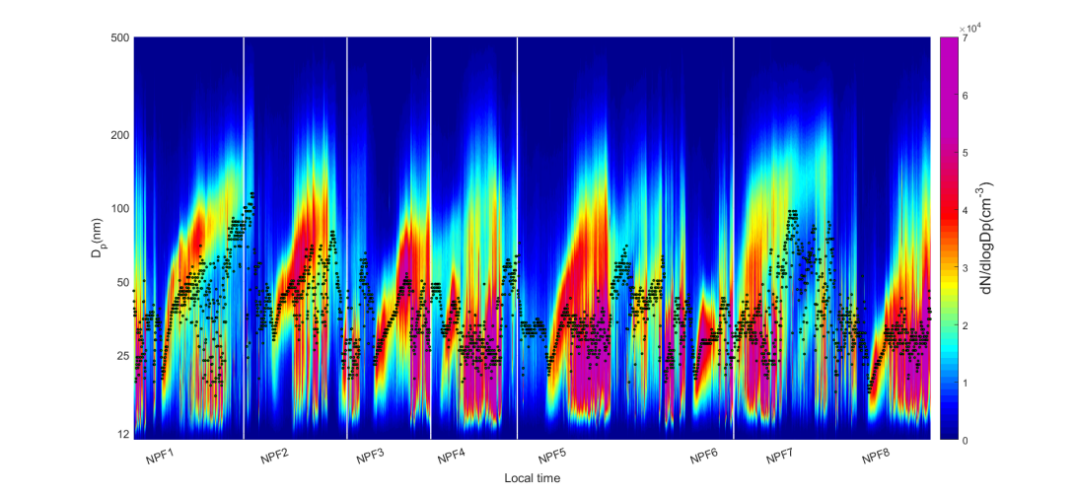
**

**Fig. S15. GMD changes during eight NPF events. Black dots show the geometric mean distribution, and the color map shows the particle number size distribution (legend on the right).**

Using the log-normal fit [40], we obtain the geometric mean distribution (Fig. S15). The beginning part of each NPF event starts with a clean background. Soon after, a banana-shaped growth of newly formed particles appears, with a burst in size in the range from 11–25 nm, followed by quick growth to the Aitken mode. However, when the wind direction changed, incoming traffic emission particles acted as large sources for the size distribution below 50 nm, causing a noticeable decrease in the fitting results. Therefore, selected were the beginning part of every event to determine the growth rate (11–25 nm). This is why distinguishing noise from signals is a challenge in the lognormal fitting method.

**4.2 Summary of cases and parameters.**

**Table S2. Summary of selected NPF cases from the Beijing site.**

| Event | NPF Start Time  (Local time) | Duration (min) | GR  (nm/h) |
| --- | --- | --- | --- |
| Case 1 | 07-Aug 09:00 | 900 | 3.8595 |
| Case 1 | 23-Aug 11:30 | 690 | 3.0305 |
| Case 1 | 05-Sep 11:00 | 540 | 2.5786 |
| Case 1 | 06-Sep 11:00 | 360 | 3.85374 |
| Case 1 | 17-Sep 10:30 | 630 | 3.04588 |
| Case 1 | 19-Sep 10:30 | 510 | 4.24992 |
| Case 2 | 20-Sep 15:30 | 690 | 2.32744 |
| Case 1 | 22-Sep 10:30 | 510 | 2.26567 |
| Case 2 | 08-Jun 09:36 | 760 | 4.08797 |
| Case 1 | 29-Aug 10:19 | 840 | 5.33655 |
| Case 1 | 04-Sep 10:04 | 950 | 6.30803 |
| Case 1 | 15-Sep 12:00 | 720 | 5.9282 |
| Case 1 | 16-Sep 10:18 | 900 | 5.33655 |
| Case 1 | 21-Sep 10:48 | 430 | 5.66646 |
| Case 2 | 02-Oct 10:46 | 920 | 5.27576 |
| Case 1 | 06-Oct 12:01 | 790 | 5.97616 |
| Case 1 | 08-Oct 10:46 | 680 | 5.23006 |
| Case 1 | 11-Oct 09:36 | 960 | 5.9282 |
| Case 1 | 20-Jan 09:36 | 648 | 5.84309 |
| Case 1 | 25-Jan 09:21 | 720 | 2.83292 |
| Case 1 | 07-Feb 08:52 | 792 | 4.17077 |
| Case 2 | 12-Mar 09:36 | 763 | 5.33655 |
| Case 1 | 13-Mar 10:19 | 676 | 5.6954 |
| Case 1 | 21-Mar 09:36 | 662 | 5.9282 |
| Case 2 | 22-Mar 09:07 | 604 | 2.64268 |
| Case 1 | 23-Mar 09:16 | 465 | 5.04614 |
| Case 2 | 25-Mar 09:49 | 706 | 3.48151 |
| Case 1 | 31-Mar 10:19 | 432 | 4.24542 |

**Table S3. Same as Table S2, for the SGP site.**

| Event | NPF Start Time  (Local time) | Duration (min) | GR  (nm/h) |
| --- | --- | --- | --- |
| Case 1 | 19-Apr 10:55 | 980 | 2.92705 |
| Case 1 | 20-Apr 13:25 | 580 | 2.71286 |
| Case 1 | 21-Apr 10:05 | 835 | 4.32759 |
| Case 2 | 24-Apr 12:40 | 500 | 1.00527 |
| Case 1 | 02-May 15:55 | 740 | 7.68845 |
| Case 2 | 03-May 10:20 | 960 | 6.58349 |
| Case 1 | 07-May 08:30 | 794 | 1.98896 |
| Case 1 | 08-May 10:30 | 70 | 7.65423 |
| Case 1 | 10-May 12:35 | 685 | 2.3251 |
| Case 1 | 11-May 12:40 | 675 | 3.70208 |
| Case 2 | 12-May 09:25 | 860 | 3.71433 |
| Case 1 | 13-May 09:05 | 780 | 3.61139 |
| Case 1 | 14-May 09:55 | 535 | 3.76455 |
| Case 1 | 20-May 09:10 | 920 | 13.1736 |
| Case 1 | 21-May 09:15 | 930 | 2.41956 |

**Table S4. Same as Table S2, for the HYY site.**

| Event | NPF Start Time  (Local time) | Duration (min) | GR (nm/h) |
| --- | --- | --- | --- |
| Case 1 | 07-Mar 12:00 | 660 | 2.85825 |
| Case 2 | 09-Mar 13:30 | 980 | 0.985414 |
| Case 2 | 11-Mar 13:30 | 960 | 3.93241 |
| Case 1 | 15-Mar 12:29 | 570 | 2.27674 |
| Case 1 | 21-Mar 09:29 | 890 | 2.20063 |
| Case 2 | 22-Mar 11:30 | 330 | 8.98257 |
| Case 1 | 24-Mar 13:00 | 790 | 4.26832 |
| Case 1 | 26-Mar 13:30 | 930 | 4.9823 |
| Case 1 | 27-Mar 10:30 | 450 | 3.23442 |
| Case 1 | 28-Mar 10:30 | 690 | 6.40944 |
| Case 1 | 29-Mar 12:29 | 920 | 5.73761 |
| Case 1 | 30-Mar 10:59 | 900 | 2.74723 |
| Case 2 | 31-Mar 10:30 | 690 | 2.34192 |
| Case 1 | 01-Apr 10:30 | 960 | 1.23204 |
| Case 2 | 04-Apr 10:00 | 940 | 2.94578 |
| Case 1 | 05-Apr 10:59 | 920 | 5.5036 |
| Case 1 | 06-Apr 10:00 | 910 | 2.27012 |

**Appendix B**

**Molecular Dynamics Simulation:**

The Reactive Force Field (ReaxFF) method was adopted in this simulation, and opensource software LAMMPS was used. LAMMPS is a classic molecular dynamics code that models ensembles of particles in a liquid, solid, or gaseous state. It can model atomic- and cluster-level systems using different force fields and boundary conditions. The time step of our simulation was 0.25 fs. The total step number for each simulation case is 16 million, achieving a simulation time of 4 ns. In the ReaxFF method, the energy equation is written in the following form. The simulated molecular dynamics using the parameters from Zhang’s [41] work can accurately demonstrate homogeneous nucleation:

$$E_{system}=E_{bond}+E_{over}+E_{under}+E_{val}+E_{pen}$$

$+E_{tors}+E_{conj}+E_{H-bond}+E_{vdWaals}+E_{Coulomb}$ . (1)

In the equation, $E_{bond}$ stand for the bond energy. $E_{over}$ and $E_{under}$ stands for the over and under coordination term. $E_{val}$ and $E_{pen}$ is the valency angle term. $E_{tors}$ and $E_{conj}$ is four-body interaction term,$E_{H-bond}$ is the hydrogen bond term, $E_{vdWaals}$ is the Van der Waals term. $E_{Coulomb}$ is the Coulomb term. To get the coulomb term, the charge distribution for the simulation system was updated by the EEM method at each timestep. Besides, the force field parameters from Zhang’s work was used in the simulation. To realize the ununiform temperature distribution, several controlled zones and transition zones were created in the simulation region. During the simulation, Berendsen thermostat with different temperature was imposed to each controlled zone. Transition zones are placed in gaps between controlled zones. In these zones, NVE ensemble is adapted.

In our simulation, the temperature perturbation was quantified by the standard deviation of temperature. For the status of low and high perturbation, the value is 3.06% and 1.02% respectively. The link between turbulence and temperature fluctuation is based on turbulence has the impact on the temperature distribution the quantitative link hasn’t been built yet. To realize the ununiform temperature distribution, several controlled zones and transition zones were created in the simulation region. During the simulation, Berendsen thermostat with different temperature was imposed to each controlled zone. Transition zones are placed in gaps between controlled zones. In these zones, NVE ensemble is adapted. The timestep of single simulation in the opensource software LAMMPS was set to be 0.25 fs. The total step number for each simulation case is 16 million so that a simulation time of 4 ns was achieved.

**References**

1. Weber RJ, Marti JJ and Mcmurry PH *et al.* Measurements of new particle formation and ultrafine particle growth rates at a clean continental site. *J Geophys Res Atmos* 1997; **102**: 4375-85.

2. Verheggen B and Mozurkewich M. Determination of nucleation and growth rates from observation of a SO2 induced atmospheric nucleation event. *J Geophys Res Atmos* 2002; **107**: AAC-1-AAC 5-12.

3. Komppula M Dal Maso and M Lihavainen H *et al.* Comparison of new particle formation events at two locations in northern Finland. *Boreal Environment Research* 2003; **8**: 395-404.

4. Dal Maso, M Sogacheva and L Aalto *et al.* Aerosol size distribution measurements at four Nordic field stations: identification, analysis and trajectory analysis of new particle formation bursts. *Tellus B* 2007; **59**: 350–61.

5. Mochida M, Miyakawa T and Takegawa N *et al.* Significant alteration in the hygroscopic properties of urban aerosol particles by the secondary formation of organics. *Geophys Res Lett* 2008; **35**: 196-9.

6. Asmi E, Frey A and Virkkula A *et al.* Hygroscopicity and chemical composition of Antarctic sub-micrometre aerosol particles and observations of new particle formation. *Atmos Chem Phys* 2009; **10**: 4253-71.

7. Cheung HC, Morawska L and Ristovski ZD. Observation of new particle formation in subtropical urban environment. *Atmos Chem Phys* 2010; **10**: 3823-33.

8. Jeong CH, Evans GJ and Mcguire ML *et al.* Particle formation and growth at five rural and urban sites. *Atmos Chem Phys* 2010; **10**: 7979-95.

9. Kalafutpettibone AJ, Wang J and Eichinger WE *et al.* Size-resolved aerosol emission factors and new particle formation/growth activity occurring in Mexico City during the MILAGRO 2006 Campaign. *Atmos Chem Phys* 2011; **11**: 8861-81.

10. Zhang YM, Zhang XY and Sun JY *et al.* Characterization of new particle and secondary aerosol formation during summertime in Beijing, China. *Tellus B* 2011; **63**: 382-94.

11. Du J. Aerosol Size Spectra and Particle Formation Events at Urban Shanghai in Eastern China. *Aerosol Air Qual Res* 2012; **12**: 1362-72.

12. Hirsikko A, Vakkari V and Tiitta P *et al.* Characterisation of sub-micron particle number concentrations and formation events in the western Bushveld Igneous Complex, South Africa. *Atmos Chem Phys* 2012; **12**: 3951-67.

13. Kuang C, Chen M and Zhao J *et al.* Size and time-resolved growth rate measurements of 1 to 5 nm freshly formed atmospheric nuclei. *Atmos Chem Phys* 2012; **12**: 3573-89.

14. Hamed A, Joutsensaari J and Mikkonen S *et al.* Nucleation and growth of new particles in Po Valley, Italy. *Atmos Chem Phys* 2013; **7**: 263-9.

15. Han Y, Iwamoto Y and Nakayama T. Observation of new particle formation over a mid-latitude forest facing the North Pacific. *Atmos Environ* 2013; **64**: 77-84.

16. Hakala S, Alghamdi MA and Paasonen P *et al.* New particle formation, growth and shrinkage at a ruralbackground site in western Saudi Arabia. *Atmos Chem Phys* 2019;**19**: 10537–10555.

17. Dada L, Chellapermal R and Buenrostro Mazon S *et al.* Refined classification and characterization of atmospheric new particle formation events using air ions. *Atmos Chem Phys* 2018;**18**: 17883–17893.

18. Hodshire AL, Lawler MJ and Zhao J *et al.* Multiple new-particle growth pathways observed at the US DOE Southern Great Plains field site. *Atmos Chem Phys* 2016; **16**: 9321-48.

19. Ehn M, Kleist E and Junninen H *et al.* Gas phase formation of extremely oxidized pinene reaction products in chamber and ambient air. *Atmos Chem Phys* 2012; **12**: 5113-27.

20. Yan C, Nie W and Petäjä M *et al.* Source characterization of highly oxidized multifunctional compounds in a boreal forest environment using positive matrix factorization. *Atmos Chem Phys* 2016; **16**: 1-31.

21. Yan C, Nie W and Äijälä M *et al.* Source characterization of highly oxidized multifunctional compounds in a boreal forest environment using positive matrix factorization. *Atmos Chem Phys* 2016; **16**: 1-31.

22. Dada L, Ylivinkka I and Baalbaki R *et al.* Sources and sinks driving sulphuric acid concentrations in contrasting environments: implications on proxy calculations. *Atmos Chem Phys Discuss* 2020; https://doi.org/10.5194/acp-2020-155

23. Donahue NM, Ortega IK and Chuang W *et al.* How do organic vapors contribute to new-particle formation? *Faraday Discuss* 2013; **165**: 91-104.

24. Lee SH, Gordon H and Yu H *et al.* New Particle Formation in the Atmosphere: From Molecular Clusters to Global Climate. *J Geophys Res Atmos* 2019; **124**: 7098– 7146

25. Zhang J, Chen Z and Lu Y *et al.* Observations of New Particle Formation, Subsequent Growth and Shrinkage during Summertime in Beijing. *Aerosol Air Qual Res* 2016; **16**: 1591-602.

26. Bianchi F, Kurten T and Riva M *et al.* Highly Oxygenated Organic Molecules (HOM) from Gas-Phase Autoxidation Involving Peroxy Radicals: A Key Contributor to Atmospheric Aerosol. *Chem Rev* 2019; **119**: 3472-509.

27. Zhang JS, Chen ZY and Lu YH *et al.* Observations of New Particle Formation, Subsequent Growth and Shrinkage during Summertime in Beijing. *Aerosol Air Qual Res* 2016; **16**: 1591-602.

28. Zheng J, Hu M and Zhang R *et al.* Measurements of gaseous H2SO4 by AP-ID-CIMS during CAREBeijing 2008 Campaign. *Atmos Chem Phys* 2011; **11**: 7755-65.

29. Wang ZB, Hu M and Pei XY *et al.* Connection of organics to atmospheric new particle formation and growth at an urban site of Beijing. *Atmos Environ* 2015; **103**: 7-17.

30. Chen M, Titcombe M and Jiang J et al. Acid-base chemical reaction model for nucleation rates in the polluted atmospheric boundary layer. Proc Natl Acad Sci USA 2012; **109**: 18713-8.

31. Rönkkö T, Kuuluvainen H and Karjalainen P *et al.* Traffic is a major source of atmospheric nanocluster aerosol. Proc Natl Acad Sci USA 2017; **114**: 7549.

32. Wang Y, Zhang F and Li Z *et al.* Enhanced hydrophobicity and volatility of submicron aerosols under severe emission control conditions in Beijing. *Atmos Chem Phys* 2017; **17**: 5239–5251.

33. Fu YL, Yu GR and Sun XM *et al.* Depression of net ecosystem CO 2 exchange in semi-arid Leymus chinensis steppe and alpine shrub. *Agric For Meteorol* 2006; **137**: 234-44.

34. Isaac P, Cleverly J and Mchugh I *et al.* OzFlux data: network integration from collection to curation. *Biogeosciences* 2017; **14**: 1-41.

35. Kulmala M, Petaja T and Nieminen T *et al.* Measurement of the nucleation of atmospheric aerosol particles. *Nat Protoc* 2012; **7**: 1651-1667.

36. Leino K, Lampilahti J and Poutanen P *et al.* Vertical profiles of sub-3&amp;thinsp;nm particles over the boreal forest. *Atmos Chem Phys* 2019; **19**: 4127-38.

37. Wehner B, Siebert H and Ansmann A *et al.* Observations of turbulence-induced new particle formation in the residual layer. *Atmos Chem Phys* 2010; **10**: 4319-30.

38. Olenius T, Pichelstorfer L and Stolzenburg D *et al.* Robust metric for quantifying the importance of stochastic effects on nanoparticle growth. *Sci Rep* 2018; **8(1)**: 14160.

39. Wei W, Zhang H and Wu B *et al.* Intermittent turbulence contributes to vertical dispersion of PM2.5 in the North China Plain: cases from Tianjin. *Atmos Chem Phys* 2018; **18**: 12953-67.

40. Hussein T, Dal Maso M and Petaja T *et al.* Evaluation of an automatic algorithm for fitting the particle number size distributions. [*Boreal Env Res*](http://www.borenv.net/) 2005; **10(5)**: 337-55.

41. Zhang WW and van Duin ACT. Second-generation ReaxFF water force field: improvements in the description of water density and OH-anion diffusion. *J Phys Chem B* 2017; **121**: 6021–6032.
